# LSD1 deletion decreases exosomal PD-L1 and restores T-cell response in gastric cancer

**DOI:** 10.1186/s12943-022-01557-1

**Published:** 2022-03-16

**Authors:** Dan-Dan Shen, Jing-Ru Pang, Ya-Ping Bi, Long-Fei Zhao, Yin-Rui Li, Li-Juan Zhao, Ya Gao, Bo Wang, Ning Wang, Liuya Wei, Huiqin Guo, Hong-Min Liu, Yi-Chao Zheng

**Affiliations:** 1 Key Laboratory of Advanced Drug Preparation Technologies, Ministry of Education of China, Key Laboratory of Henan Province for Drug Quality and Evaluation, Institute of Drug Discovery and Development, School of Pharmaceutical Sciences, Zhengzhou University, 100 Kexue Avenue, Henan 450052 Zhengzhou, China; 2 State Key Laboratory of Esophageal Cancer Prevention & Treatment, Academy of Medical Sciences, Zhengzhou University, 100 Kexue Avenue, Zhengzhou, Henan China; 3 The School of Chinese Medicine, The University of Hong Kong, Pokfulam, Hong Kong, China; 4 School of Pharmacy, Weifang Medical University, Weifang, Hebei China; 5 Thoracic Department, Beijing Shijitan Hospital, Capital Medical University, Beijing, China

**Keywords:** LSD1, PD-L1, Gastric cancer, Exosomes, Tumor immunity

## Abstract

**Background:**

Histone lysine-specific demethylase 1 (LSD1) expression has been shown to be significantly elevated in gastric cancer (GC) and may be associated with the proliferation and metastasis of GC. It has been reported that LSD1 repressed tumor immunity through programmed cell death 1 ligand 1 (PD-L1) in melanoma and breast cancer. The role of LSD1 in the immune microenvironment of GC is unknown.

**Methods:**

Expression LSD1 and PD-L1 in GC patients was analyzed by immunohistochemical (IHC) and Western blotting. Exosomes were isolated from the culture medium of GC cells using an ultracentrifugation method and characterized by transmission electronic microscopy (TEM), nanoparticle tracking analysis (NTA), sucrose gradient centrifugation, and Western blotting. The role of exosomal PD-L1 in T-cell dysfunction was assessed by flow cytometry, T-cell killing and enzyme-linked immunosorbent assay (ELISA).

**Results:**

Through in vivo exploration, mouse forestomach carcinoma (MFC) cells with LSD1 knockout (KO) showed significantly slow growth in 615 mice than T-cell-deficient BALB/c nude mice. Meanwhile, in GC specimens, expression of LSD1 was negatively correlated with that of CD8 and positively correlated with that of PD-L1. Further study showed that LSD1 inhibited the response of T cells in the microenvironment of GC by inducing the accumulation of PD-L1 in exosomes, while the membrane PD-L1 stayed constant in GC cells. Using exosomes as vehicles, LSD1 also obstructed T-cell response of other cancer cells while LSD1 deletion rescued T-cell function. It was found that while relying on the existence of LSD1 in donor cells, exosomes can regulate MFC cells proliferation with distinct roles depending on exosomal PD-L1-mediated T-cell immunity in vivo.

**Conclusion:**

LSD1 deletion decreases exosomal PD-L1 and restores T-cell response in GC; this finding indicates a new mechanism with which LSD1 may regulate cancer immunity in GC and provides a new target for immunotherapy against GC.

**Supplementary Information:**

The online version contains supplementary material available at 10.1186/s12943-022-01557-1.

## Background

LSD1 was characterized in 2004 as the first histone demethylase [1] that specifically removes mono- and di-methylation of histone H3K4 with the corepressor for RE1-silencing transcription factor (CoREST) complex as well as with the androgen receptor, resulting in different transcriptional regulation in distinct contexts [2–4]. Because of its involvement in a wide range of biological processes, including cell development, differentiation, growth, migration, and stemness [5–9], LSD1 acts as an oncogene in diverse cancers [10–15]. In our previous studies, LSD1 expression was shown to be elevated in GC and promoted the proliferation and metastasis of GC, while treatment with its inhibitors suppressed the growth, invasion, and migration of GC cells [16–19]. LSD1 can also promote the tumor development process by regulating the tumor microenvironment mediated by natural killer (NK) cells [20], macrophage polarization [21]. Inhibition of LSD1 stimulates antitumor immunity and enhances antitumor efficacy of programmed cell death 1/PD-L1 (PD-1/PD-L1) blocker in breast cancer and melanoma [22, 23]. However, functions of LSD1 in tumor immune microenvironment of GC still remain unclear. Although PD-L1 upregulation occurs in approximately 40% of gastroesophageal cancers and the United States Food and Drug Administration (FDA) has approved pembrolizumab and nivolumab for patients with GC and completely resected esophageal or gastroesophageal junction cancer [24–26], preliminary clinical data with single-agent treatment with PD-1/PD-L1 inhibitors showed low response rates of only 22%-27% for metastatic gastroesophageal cancer patients with PD-L1^+^ expression [27].

In our exploration of LSD1 function in immune microenvironment of GC, we found, by comparing the function of LSD1 in immunodeficient and normal mice, that LSD1 maintained tumor growth by repressing T-cell activity in GC. As LSD1 deletion reduced the total expression of PD-L1 but maintained its expression level on the membrane of GC cells. Exosomes, a class of small vesicles of 30–150 nm in diameter secreted by normal cells and cancer cells, attracted us due to the reports that exosomes can harbor PD-L1 to suppress anticancer immunity [28–33]. Herein, PD-L1 was identified as a cargo in GC cell-derived exosomes, and GC cells were found to maintain cell membrane PD-L1 as well as reduce the secretion of exosomal PD-L1 when LSD1 was abrogated. Meanwhile, LSD1 deletion can restore the killing function of T cells in the microenvironment of GC by decreasing the amount of PD-L1 in exosomes as well as by inhibiting PD-L1 transportation to other cancer cells through exosomes, thereby offsetting its immunosuppressive function. These results indicated a new mechanism by which LSD1 suppressed tumor immunity in GC and may provide a new strategy for the immunotherapy against GC by using LSD1 as a treatment target.

## Methods

### Cell culture

Human gastric cancer cells AGS, BGC-823, HGC-27, MGC-803, MKN-45, NCI-N87 as well as MFC cell lines were purchased from National Cell Resource Center. All the cells were identified by short tandem repeat (STR). All the human cells were cultured in Roswell Park Memorial Institute 1640 medium (01–100-1ACS, BI, Israel) supplemented with 10% (v/v) fetal bovine serum (FBS) (01–052-1, BI, Israel) and cultured in an incubator at 37℃ with 5% CO_2_. MFC cells were cultured in Dulbecco’s modified Eagle medium (01–051-1ACS, BI, Israel) supplemented with 10% FBS (01–052-1, BI, Israel).

### Establishment of LSD1 KO cell lines

Single guide RNA (sgRNA) oligonucleotides were cloned into U6-sgRNA-EF1a-Cas9-FLAG-P2A-puro (Human)/ U6-sgRNA-SFFV-Cas9-FLAG-P2A-mCherry (Mouse) and packaged as lentiviruses (Genechem, Shanghai, China). The viruses were subjected to the human GC cell lines BGC-823, MGC-803 and MFC cells from mice according to the published protocol [22]. The medium was changed to remove the virus 12 h after transfection, the transfection efficiency was tested after 72 h of culture, then dilution method was used to pick single clones. KO clones were identified by the Western blot. The LSD1 KO sgRNA sequence were: human CCGGCCCTACTGTCGTGCCT, mouse CCTGAGAGGTCATTCGGTCA.

### Isolation of exosomes by ultracentrifugation

Cells were cultured with serum-containing medium for 24 h and then changed to serum-free medium for another 36 h. Then, the conditional medium was collected and centrifuged at 5000 g for 30 min to wipe off the cell debris, followed by centrifugation at 10,000 g for 30 min to wipe off large vacuoles. Exosomes were collected by ultracentrifugation at 100,000 g for 2 h and subsequently washed with phosphate buffer solution (PBS), followed again by centrifugation at 100,000 g for 2 h. The pellet after centrifugation was collected as exosomes, and then resuspended in PBS and stored at -80 °C until use. All exosomes were filtered with a 0.22 μm filter before use. Unless otherwise specified, exosomes were all obtained by differential ultracentrifuge. The concentration of exosomes was quantified using a bicinchoninic acid (BCA) assay kit (PC0020, Solarbio, China).

### Isolation of exosomes by sucrose gradient centrifugation

Sucrose gradient fractionation was conducted as previously described with minor modifications [34]. Briefly, exosomes obtained by differential ultracentrifugation were centrifuged again at 100,000 g for 2.5 h and fractions were collected as follows: 10–16% (F1), 22–28% (F2), 34–40% (F3), 46–52% (F4), 58–64% (F5), and 70–82% (F6) sucrose solutions. The fractions were diluted 1:100 in PBS and centrifuged at 100,000 g for another 2.5 h to pellet any extracellular vesicles (EVs). Pellets were resuspended in PBS for the Western blotting. Samples were denatured with a loading buffer and analyzed with sodium dodecyl sulfate–polyacrylamide gel electrophoresis (SDS-PAGE) by volume normalization for further Western blotting analysis with exosomal marker protein antibodies, anti-CD9 (13403S, CST, USA), anti-CD63 (ab59479, abcam, USA), and anti-ALIX (2171S, CST, USA).

### NTA of exosomes

The size distribution and concentration of isolated exosomes were measured using a NanoSight NS300 instrument (Malvern Instruments, Ltd., Malvern, UK). The data were analyzed using NTA software (NTA version 2.3 build 0017, Malvern Instruments Ltd.). To perform the measurement, exosome samples were filtered using 0.22 μm-filter membranes and diluted 10- to 100-fold in PBS to make the number of particles in the field of view about 100 per frame.

### TEM of exosomes

About 30 μL exosomes sample was dropped on carbon-plated support film copper mesh which was placed on the sealing film and let it stay for 2–5 min. Then use a pointed filter paper to absorb the excess solution from the edge, and take it to the filter paper to stay about 10 min. After support film was dried, a drop of uranyl acetate dye solution was dripped and dyed for 90 s. Then the excess dye solution was absorbed and support film was clamped on the filter paper and dried for 3 h to observe.

### Detection of exosomal PD-L1 using flow cytometry

Conditioned medium of BGC-823, MGC-803 and their corresponding LSD1 KO cells were incubated with CD63 exosome capture beads (ab239686, abcam, USA) in the dark overnight at room temperature. Then, the mixture was washed with 1 mL PBS twice, and the supernatant was discarded. After that, exosomes with beads were suspended in PE-conjugated anti-human PD-L1 antibody (1:5) (557924, BD, USA) for 40 min at 4℃. PE-conjugated IgG (556650, BD, USA) was set as negative control, then washed with PBS. Finally, the exosomes were analyzed by flow cytometry (BD, USA) after resuspension with 500 μL staining buffer. The whole process was protected from exposure to light.

### Detection of membrane PD-L1

Cells were harvested and washed twice with 1.0 mL PBS with 2% FBS, and then mixed gently. After centrifugation at 400 g for 5 min at 4˚C, the supernatant was discarded, and the process was repeated. Cells were suspended with PE-conjugated anti-human PD-L1 antibody (1:5) (557924, BD, USA) for 40 min at 4℃. Then, cells were washed twice with PBS containing 2% FBS. Finally, the stained cells were analyzed by flow cytometry after resuspension with 500 μL staining buffer. The whole process was protected from light.

### Exosomes PD-1/PD-L1 binding assay

Human PD-1-Fc fusion protein (5 μg/ml) (10377-H02H, SinoBiological, China) was coated on high protein-binding 96-well plates for 1 h at 37℃ and BSA (5 μg/ml) (A8020, Solarbio, China) was used as a control. Samples were blocked with 5% BSA for 2 h at room temperature. Simultaneously, exosomes were strained with PKH26 red fluorescent labeling kit (MINI26-1KT, Sigma, Germany) for 5 min then subjected to ultracentrifugation at 100,000 g for 2 h to remove superfluous PKH26, and then resuspended in PBS. The plate was washed three times with PBS and then the labeled exosomes were added to the plates for 2 h at room temperature. After washing three times, samples were analyzed by fluorescence microscopy. The whole process was protected from light [35].

### Exosomes fusion assay

Cells were seeded into 24-well plates with glass coverslips and allowed to reach 50% confluence. Meanwhile, exosomes were stained with PE-conjugated anti-human PD-L1 antibody (1:5) (557924, BD, USA) for 40 min, then the redundant antibody were washed with PBS and removed by ultracentrifugation. The exosomes were added to the plate and incubated for 24 h at 37℃ and 5% CO_2_. Cells were washed with PBS three times and fixated with 4% neutral formalin. After washing with PBS three times, cells were incubated with 3,3’-dioctadecyloxacarbocyanine perchlorate (DIO 5 μmol/L) (C1038, Beyotime, China) and 4',6-diamidino-2-phenylindole (DAPI, 100 ng/mL) (BS130A, Biosharp, China) for 10 min. Cells were washed two times with PBS and placed on the slide with anti-fluorescence quenching agent. The fluorescence of cells was observed under a confocal microscope (Nikon, Japan) using a corresponding channel.

### Peripheral blood mononuclear cell (PBMC) separation and activation

Blood was obtained from healthy donors and human lymphocytes were separated using lymphocyte separation medium (P8610, Solarbio, China) as described by the manufacturer’s protocol. Then, the lymphocytes were cultured in Roswell Park Memorial Institute 1640 medium (01–100-1, BI, Israel) supplemented with 10% FBS in an incubator at 37℃ and 5% CO_2_. The lymphocytes were then activated with anti-CD3/CD28 beads (11161D, Thermo Fisher, USA) for 24 h at 37℃ and 5% CO_2_.

### T-cell proliferation assay

For the T-cell proliferation study, PBMC were stained with 5, 6-carboxyfluorescein diacetate, succinimidyl ester (CFSE) (5 μg/mL) (C1031, Beyotime, China) for 24 h. Then, anti-CD3/CD28 beads and exosomes were added to the cells and incubated for 3 days at 37℃ and 5% CO_2_. After that, T cells were stained with APC-CD3 antibody (300,312, Biolegend, USA). Consequently, cells were analyzed by flow cytometry after rinsing with PBS. The whole process was protected from light. The data were analyzed using FlowJo (FlowJo 10.4, BD, USA).

### T-cell cytotoxicity assay

After the indicated treatment, cells were seeded into pre-coated 96-well plate at a density of 2,000 cells per well. Then, activated lymphocytes were added to the wells at 10:1 ratio and incubated in 37℃ for 4 days. After that, cells were washed with PBS and incubated with DAPI (100 ng/mL) for 10 min. Finally, cells were washed with PBS and counted with high content analyzer (Thermo Fisher, USA).

### Cellular PD-1/PD-L1 binding assay

Cells were seeded into 24-well plates with glass coverslips. Then, exosomes (10 μg/mL) were added to the cells and incubated at 37℃ for 48 h. After that, cells were washed and fixed with 4% neutral formaldehyde for 20 min at room temperature, and the human PD-1 Fc fusion protein (5 μg/ml) were added to the wells and incubated at 4℃ overnight, followed by anti-rabbit Alexa Fluor 488 dye conjugated antibody (ZF-0511, Origene, China) (1:200) staining for 2 h at room temperature. Finally, DAPI (100 ng/mL) was applied for another 10 min and anti-fluorescence quencher (S2100, Solarbio, China) was used to seal the slides. Cells were analyzed by confocal microscopy (Nikon, Japan). The whole process was protected from light.

### ELISA

Supernatants from PBMC which was activated with anti-CD3/CD28 beads as well as treated with exosomes from BGC-823 cells (B-EXO) or BGC-823 LSD1 KO cells (B KO-EXO) were tested for interleukin 2 (IL-2), interferon-gamma (IFN-γ) or tumor necrosis factor alpha (TNFα) concentration by ELISA (88–7025-22, 88–7316-22, 88–7346-22, Thermo Fisher, USA). The IL-2, IFN-γ, or TNFα concentrations were calculated with reference to standard curves and expressed as ng/mL. Mice tumor tissues were broken by tissue homogenizer in PBS and the supernatants were tested for IL-2, IFN-γ concentration using ELISA (88–7024-22, 88–7314-22, Thermo Fisher, USA). The concentrations were calculated with reference to standard curves and normalized to the MFC group.

### Western blot

Cells were collected and lysed using radio immunoprecipitation assay (RIPA) lysis buffer (50 mM Tris–HCl, pH 7.5, 150 mM NaCl, 0.25% sodium deoxycholate, 0.1% nonidet P-40, 0.1% Triton X-100) with the complete proteinase inhibitor cocktail (Roche, Switzerland) for 30 min. After centrifugation at 14,000 g for 10 min at 4 °C, supernatant was collected and quantified using a BCA assay kit (PC0020, Solarbio, China). After addition of loading buffer, the cell lysis was denatured for 10 min at 100 °C for the subsequent SDS-PAGE. Then the proteins were transferred to nitrocellulose membranes (Pall, USA). Membranes were blocked with 5% skim milk at room temperature for 2 h, followed by incubation with primary antibodies at 4˚C overnight. After washing the membranes with PBST (PBS, 0.05% Tween-20) 4 times (5 min per wash), the membranes were incubated with the secondary antibody (1:5000) (peroxidase-conjugated goat anti-rabbit IgG, ZB-2301, Zsbio, China; peroxidase-conjugated goat anti-mouse IgG, ZB-2305, Zsbio, China) at room temperature for 2 h. Finally, membranes were washed with PBST 4 times (5 min per wash). The antibody-reactive bands were revealed by enhanced chemiluminescence (32209, Thermo Fisher, USA) and exposed on radiographic film.

### Real-time quantitative polymerase chain reaction (qRT-PCR)

Total RNA was extracted using Trizol regent (10296010, Thermo Fisher, USA) and then cDNA was synthesized using HiScript II Q RT SuperMix for qPCR (R223-01, Vazyme, China). qRT-PCR was performed with ChamQ Universal SYBR qPCR Master Mix (Q711-02, Vazyme, China) on the StepOne Real-Time PCR System (Life Technologies, USA). The following primers were used to detect the expression of PD-L1 and β-actin: human PD-L1, 5’-TCACTTGGTAATTCTGGGAGC-3’ (forward) and 5’-CTTTGAGTTTGTATCTTGGATGCC-3’ (reverse); β-actin, 5’-GCAAAGACCTGTACGCCAACA-3’ (forward) and 5’-TGCATCCTGTCGGCAATG-3’ (reverse).

### Immunofluorescence

After treatment, cells were washed with PBS and fixed with 4% neutral formaldehyde for 20 min at room temperature. After washing three times with PBS, the primary antibodies, ras-related GTP-binding protein 11 (RAB11) (5589 T, CST, USA) and tumor susceptibility gene 101 protein (TSG101) (ab83, abcam, USA) were added to the cells according to the required concentration and incubated at 4℃ overnight. After washing three times with PBS, cells were incubated with anti-rabbit Alexa Fluor 488 dye conjugated antibody (ZF-0511, Origene, China) or anti-mouse Alexa Fluor 594 dye conjugated antibody (ZF-0513, Origene, China) (1:200) for 2 h at room temperature. Then, cells were washed and stained with DAPI (100 ng/mL). Finally, anti-fluorescence quencher was used to seal the slide and cells were analyzed by confocal microscopy (Nikon, Japan). The whole process was protected from light.

### Ethics declarations

GC tissues and adjacent tissues were obtained from the First Affiliated Hospital of Zhengzhou University. All human tissues were collected using protocols approved by the Ethics Committee of the Zhengzhou University Health Science Center.

### Immunohistochemistry staining

Tissue specimens were fixed in 10% formalin solution and embedded in paraffin wax, then 5 μm serial sections were cut from the tissue blocks, deparaffinized in xylene, and dehydrated in a series of alcohol concentrations (75%, 85%, 95%, 100%), followed by antigen retrieval with ethylene diamine tetraacetic acid (EDTA) or citrate buffer and blocked with 5% goat serum. Tissue sections were then incubated with primary antibodies against LSD1 (ab129195, abcam, UK), CD3 (ab16669, abcam, UK), and CD8 (human, ET1606-31; mouse, 0108–7, Huabio, China,). Subsequently, tissue sections were incubated with secondary antibodies (peroxidase-conjugated goat anti-rabbit Ig, ZB-2301, Zsbio, China; peroxidase-conjugated goat anti-mouse IgG, ZB-2305, Zsbio, China) for 2 h at room temperature, and stained with DAB kit (ZL1-9018, ZSGB-BIO, China). After staining, sections were digitally scanned using the Aperio AT2 scanner (Leica Biosystems, Germany), and analyzed with Aperio image analysis workstation (Leica Biosystems, Germany) using a pathologist-trained nuclear, membranal, and nuclear & cytoplasmic algorithms. Protein expression was evaluated according to the H-Score obtained from Aperio image analysis workstation.

### Animal studies

Six to eight-week-old female mice were used for all experiments. 615 mice were purchased from Institute of Hematology, Chinese Academy of Medical Sciences. In addition, BALB/c nude mice were purchased from Jingda Laboratory Animal, Hunan, China. Before the experiments, all purchased mice were allowed one week to acclimate to housing conditions in a pathogen-free environment.

For subcutaneous tumor formation experiments, 2.5 × 10^5^/100 μL MFC or MFC LSD1 KO cells were digested and resuspended in sterilized PBS and then injected subcutaneously into 615 mice or BALB/c nude mice with 6 mice contained in each group. Tumor volume was monitored every 3 days using a digital caliper according to the formula: TV (mm^3^) = length × width^2^ × 0.5.

For in vivo studies of exosomes, 2.5 × 10^5^/100 μL MFC or MFC LSD1 KO cells/100 μL were injected subcutaneously into 615 mice as described above with 6 mice contained in each group. After the tumor volume reached about 100 mm^3^ (about one week), the mice were treated with 20 μg exosomes or PD-1 recombinant protein blockading the exosomes via intratumoral injection two times a week. After 14 days, the mice were euthanized, and the tumors were isolated and weighed.

All mice were housed in specific pathogen-free conditions and protocols were approved by the Ethics Committee of the Zhengzhou University Health Science Center.

### Tumor-infiltrating leukocyte detection by flow cytometry

Tumors were stripped and cut into 2 mm sized pieces and digested in 1 mg/mL collagenase type 4 (LS004188, Thermo Fisher, USA) and hyaluronidase (H3506, Sigma, USA). Samples were then incubated in a water bath for 60 min at 37℃, and passed through a 70 mm filter. Then the cell suspension was stained with PE-CY7-CD45 (103114, Biolegend, USA), APC-CD3 (300312, Biolegend, USA), APC-CD4 (100412, Biolegend, USA), PE-CD8a (100708, Biolegend, USA) for 40 min. Finally, the stained cells were analyzed by flow cytometry after resuspension with a staining buffer. The whole process was protected from light.

### Statistical analysis

Data analysis was performed in GraphPad Prism 9 (GraphPad Company, USA) and bar graphs indicate mean ± standard deviation (SD). Fold change is relative to control groups. Statistical significance was achieved when P value was below 0.05. Co-localization between two stained proteins were analyzed by ImageJ 1.47v (National Institutes of Health, USA). In this study, the statistical analysis was performed with Student’s *t* Test. *P < 0.05, **P < 0.01, **P < 0.001.

## Results

### LSD1 abrogation inhibits tumor growth by suppressing T-cell response in GC

**Fig. 1:**
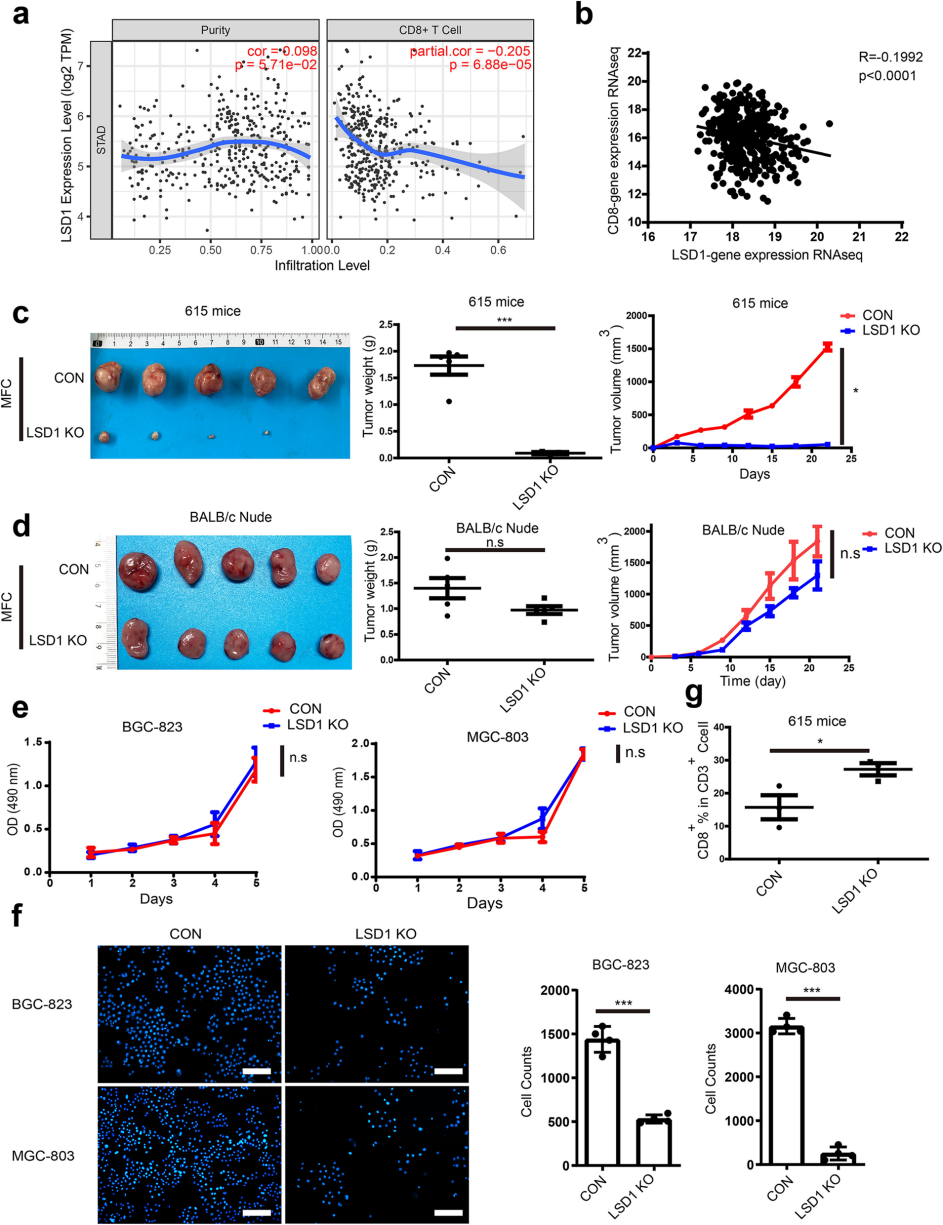
LSD1 KO can inhibit tumor growth by promoting T-cell response in GC. **a** Relationship between LSD1 and CD8 + T-cell infiltration level in GC analyzed by TIMER2.0. **b** Relationship between LSD1 and CD8 mRNA in GC using data from TCGA database (*n* = 407). **c** and **d** Images of tumors, tumor weight, and tumor volume curves of 615 mice (**c**) and BALB/c nude mice (**d**) bearing 2.5 × 10^5^ MFC cells whether LSD1 was abrogated or not (The data are presented as the mean ± SD, *n* = 6). **e** Growth curves of BGC-823 and MGC-803 cells in the presence or absence of LSD1. **f** Cell survival of BGC-823 and MGC-803 cells in the presence or absence of LSD1, treated with anti-CD3/CD28-activated T cells. Scale bar = 200 μm. g Percentage of CD8^+^ T cells in CD3^+^ infiltration cells isolated from tumors of 615 mice bearing MFC cells in the presence or absence of LSD1. The data are presented as the mean ± SD; *n* = 3; n.s, no significance, *P < 0.05; two-tailed unpaired Student’s t-test

To determine the regulation of tumor immunity by LSD1 in GC, the correlation between LSD1 and immune cell signatures were first analyzed by TIMER2.0, a platform that provides robust estimation of immune infiltration levels [36]. Results showed that LSD1 was negatively correlated with tumor infiltrating CD8^+^ T cells mostly in GC (Fig. 1a) among diverse immune cells (Supplementary Fig. 1a). Further analysis using The Cancer Genome Atlas (TCGA) database revealed that mRNA of LSD1 was negatively correlated with CD8 and CD3 mRNA expression in GC (Fig. 1b and Supplementary Fig. 1b). Hence, LSD1 was supposed to maintain tumor growth by inhibiting T-cell response. LSD1 KO cell lines were established in human GC cell lines BGC-823 and MGC-803 as well as MFC cell line (Supplementary Fig. 1c and 1d). Then the MFC and MFC LSD1 KO cells were subcutaneously inoculated into 615 mice and T-cell deficient BALB/c nude mice, respectively. After 3 weeks, tumors were dissected and weighted. The results showed that the MFC LSD1 KO group exhibited equivalent tumor weight and volume than the MFC group in BALB/c nude mice, whereas tumors in the 615 mice were almost completely eradicated in the MFC LSD KO group (Fig. 1c and d), while their body weight stayed consistent (Supplementary Fig. 1e and 1f). These results indicated that LSD1 was likely to maintain tumor growth by inhibiting T-cell response. In addition, results from co-incubation of human GC cells with anti-CD3/CD28 beads-activated T cells showed that BGC-823 and MGC-803 cells alone grew stably in vitro, whether LSD1 was knocked out or not (Fig. 1e), while these two cancer cell lines could be killed by activated T cells more easily when LSD1 was absent (Fig. 1f). By analyzing the stripped tumors from 615 mice in Fig. 1c, absence of LSD1 was resulted in increased CD8^+^ T-cell infiltration (Fig. 1g), which is consistent with results in Fig. 1a. Collectively, these findings demonstrated that LSD1 is a suppressor of T-cell response in GC, and LSD1 deletion may significantly inhibit the growth of GC cells by promoting T-cell killing ability in vitro and in vivo.

### LSD1 is negatively correlated with CD8 and positively correlated with PD-L1 in GC specimens

**Fig. 2:**
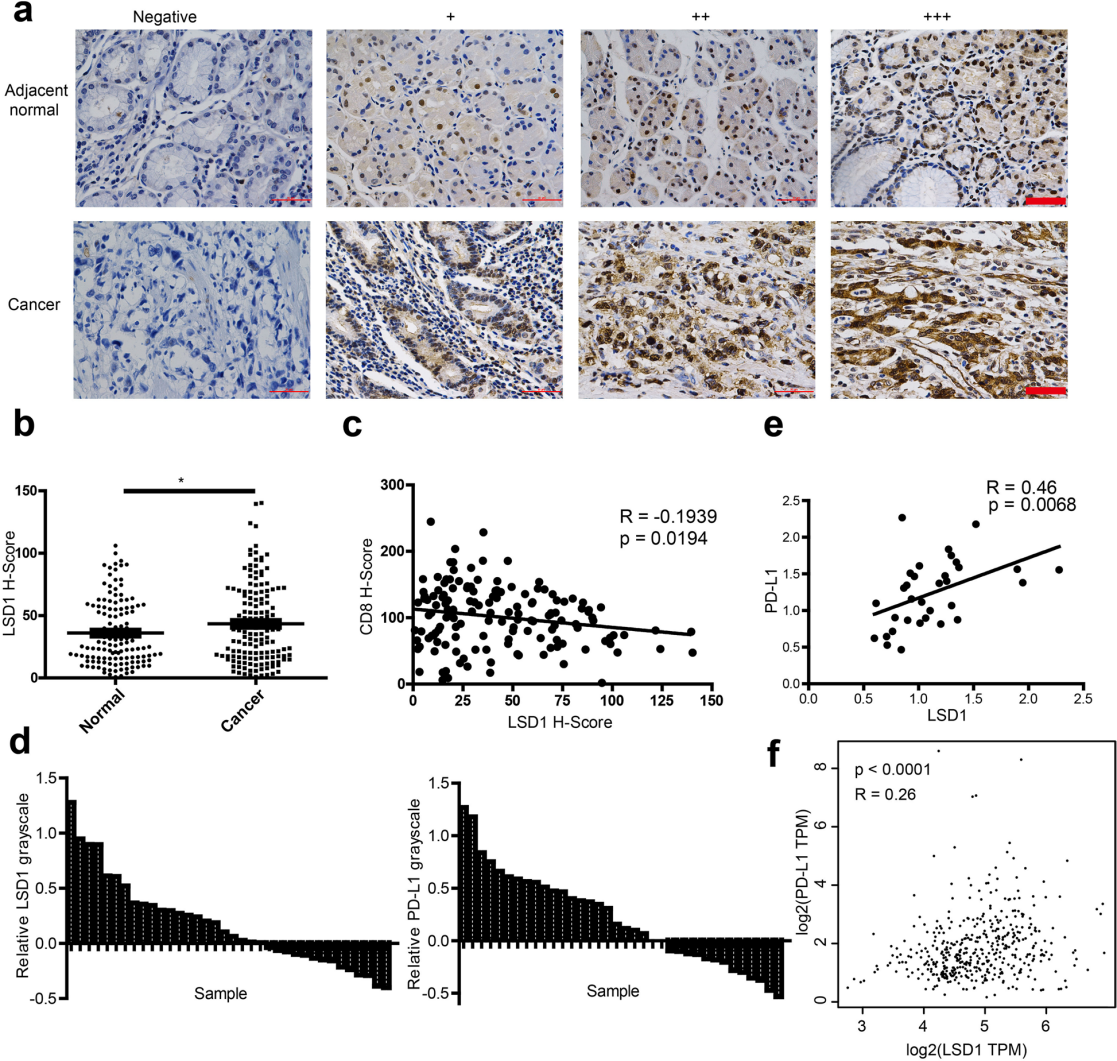
Positive correlation between expression of LSD1 and immune regulatory genes in GC specimens. **a** Representative images of LSD1 expression in normal gastric tissues and GC tissues stained by IHC. Scale bar = 50 μm. **b** Expression analysis of LSD1 in normal and cancer tissues form 145 GC patients. **c** Relationship between LSD1 and CD8 expression in cancer tissues form 145 GC patients. **d** Comparison of LSD1 and PD-L1 expression in 36 pairs of GC tissues and their corresponding adjacent normal tissues. y = (Grayscaletumor-Grayscalenormal)/ Grayscale normal. **e** Expression relationship between LSD1 and PD-L1 in 36 pairs of GC tissues. **f **Relationship between LSD1 and PD-L1 mRNA in GC using TCGA database

Tumor infiltrating CD8^+^ cytotoxic T lymphocytes are recruited to the tumor site by locally secreted chemokines. The number of cells vary by activation and proliferation or exhaustion and apoptosis of CD8^+^ T cells, which can be induced by other cells or factors in the tumor microenvironment [37, 38]. To explore how LSD1 affects the level of tumor-infiltrating CD8^+^ T cells, expressions of LSD1, CD8, and T-cell attracting chemokines, C-X-C motif chemokine 9 (CXCL9) and C-X-C motif chemokine 10 (CXCL10) were analyzed using IHC on tissue microarray (TMA) blockers constructed with our in-house GC specimens. Result in Fig. 2a and 2b suggested that LSD1 was overexpressed in GC tissues, and negatively correlated with CD8 (Fig. 2c) while CXCL9 and CXCL10 were hardly expressed in GC tissues (Supplementary Fig. 2a). As inhibition of LSD1 stimulates antitumor immunity and enhances antitumor efficacy of PD-1/PD-L1 blockers in breast cancer and melanoma [21, 22], and PD-L1 is an effective target of immunotherapy that can inhibit T-cell activation by binding PD-1 [39–41], we conjectured that LSD1 may be mainly involved in the development of GC by inhibiting the activation and proliferation of T cells through PD-L1. To explore the correlation between LSD1 and T-cell co-repressor PD-L1 in GC, 36 GC specimens were collected and subjected to further analysis. The results in Fig. 2d and Supplementary Fig. 2b suggested that the expression of LSD1 and PD-L1 in cancer tissues was higher than that in adjacent tissues, and PD-L1 was positively correlated with LSD1 (Fig. 2e). In TCGA database, PD-L1 expression was also positively correlated with LSD1 mRNA expression in GC (Fig. 2f). These results preliminary support our hypothesis that there may be a regulatory relationship between LSD1 and PD-L1.

### Inhibition of LSD1 downregulates PD-L1 in GC

**Fig. 3:**
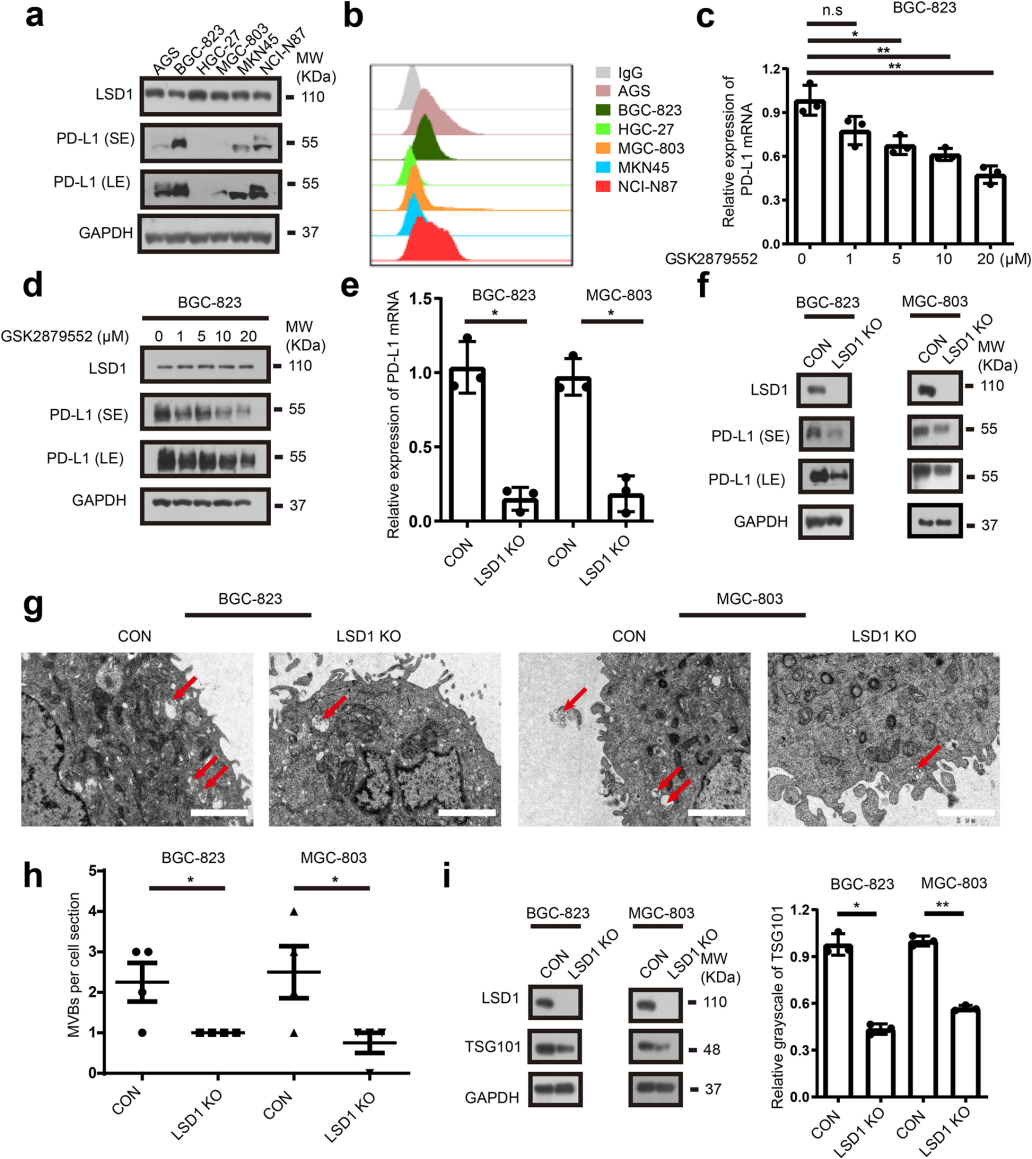
Inhibition of LSD1 downregulates PD-L1 gene levels in GC. **a** and **b** Expression of total PD-L1 (**a**) and membrane PD-L1 (**b**) in a panel of GC cell lines. **c **and** d** mRNA (**c**) and protein (**d**) expression of PD-L1 when BGC-823 cells were exposed to GSK2879552 for 5 days. **e** and **f **mRNA (**e**) and protein (**f**) expression of PD-L1 in BGC-823 and MGC-803 cells in the presence or absence of LSD1. **g** and **h** Morphology (**g**) and number (**h**) of polyvesicular corpuscles in BGC-823, MGC-803 cells in the presence or absence of LSD1. Scale bar = 2 µm (The data are presented as the mean ± SD; *n* = 4). **i** Expression of LSD1 and TSG101 in BGC-823, MGC-803 cells in the presence or absence of LSD1. The data are presented as the mean ± SD; *n* = 3; n.s, no significance, *P < 0.05; two-tailed unpaired Student’s t-test

To further explore the regulation of PD-L1 by LSD1 in GC, total PD-L1 and membrane PD-L1 in a panel of GC cells were quantified. As shown in Fig. 3a and b, there was visibly uneven expression of total PD-L1 and membrane PD-L1 across different GC cell lines. The BGC-823 cell line was selected for the following study due to its relatively high expression of PD-L1, and MGC-803 cell line was also selected due to its relatively low expression of PD-L1. To investigate the regulatory role of LSD1 on PD-L1, LSD1 was abrogated pharmacologically using LSD1 inhibitor GSK2879552 (Fig. 3c and d, Supplementary Fig. 3a) [12] or genetically using sgRNA (Fig. 3e and f). The results indicated that PD-L1 expression was downregulated both at the mRNA level and protein level when LSD1 was abrogated. Since PD-L1 is a transmembrane protein and exerts an immunosuppressive function in the extracellular region, the amount of membrane PD-L1 in GC cells when LSD1 was abrogated was also evaluated. Unexpectedly, the expression level of membrane PD-L1 did not change significantly (Supplementary Fig. 3b and 3c), which piqued our interest in the relocation of the PD-L1. Previous studies have reported that PD-L1 could be degraded through the proteasomal and lysosomal pathways, but whether LSD1 inhibitors affect the degradation pathways of PD-L1 remains to be known [42–45]. To investigate this, GC cells were treated with protein synthesis inhibitor cycloheximide (CHX) and lysosomal inhibitor chloroquine (CQ) [46]. Data in Supplementary Fig. 3d and 3e revealed that LSD1 deletion did not decrease PD-L1 degradation by proteasomes nor lysosomes. Therefore, attention on the synthesis and degradation pathways of PD-L1 was redirected to the endosomal transport pathway, as PD-L1 is able to be transported in the cytoplasm through the recovery and maturation of endosomes [42, 45]. To probe into the LSD1 function in endosomal transport, recovery endosome marker protein RAB11 and multivesicular bodies marker protein TSG101 were detected [47]. The results showed that LSD1 deletion could increase RAB11 expression (Supplementary Fig. 3f), and PD-L1 co-localized with RAB11 and TSG101 in the cytoplasm (Supplementary Fig. 3g and 3h). The SEM results also showed that the number of multivesicular bodies were decreased when LSD1 was knocked out (Fig. 3g and h). Meanwhile, TSG101 expression was decreased when LSD1 was deleted (Fig. 3i). Above results indicated that LSD1 deletion could reduce PD-L1 total expression and maintain membrane PD-L1 level as well as decrease the secretion of PD-L1 on the outside of the cells through multivesicular bodies, promoting PD-L1 recycling to the membrane in GC cells.

### LSD1 deletion downregulates exosomal PD-L1 in GC

**Fig. 4:**
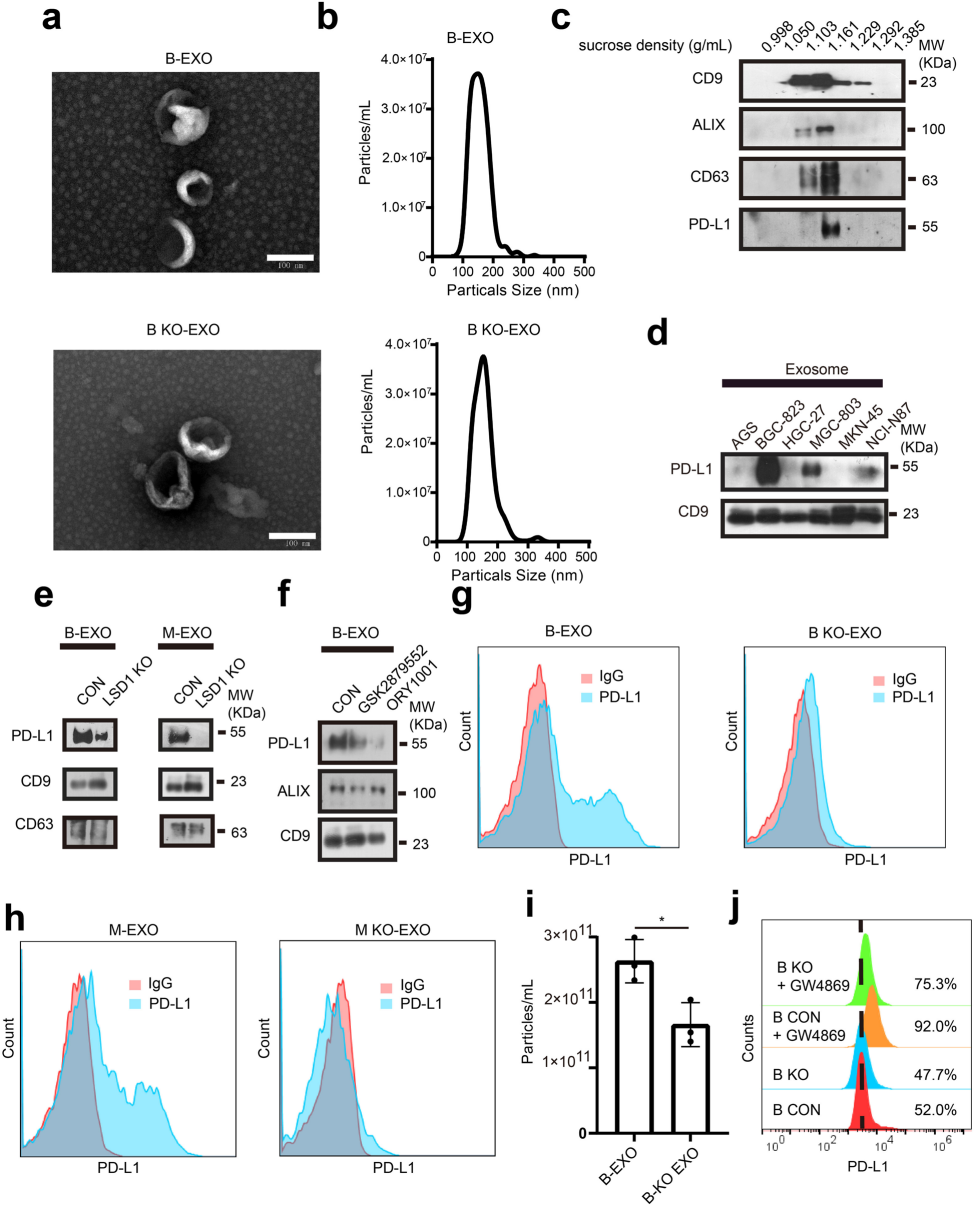
LSD1 deletion downregulates exosomal PD-L1 in GC. **a** SEM images of purified exosomes from BGC-823 cells in the presence or absence of LSD1 (B-EXO and B KO-EXO, respectively, for short). Scale bar = 100 nm. **b** Size distribution of the BGC-823 and LSD1 deleted BGC-823 cell-derived exosomes analyzed by NTA. **c **Analysis of the fractions collected after sucrose gradient centrifugation at 100,000 g; pellets obtained from ultracentrifugation of BGC-823 cells supernatants. **d** Expression of PD-L1 in a panel of GC cell-derived exosomes. **e** Expression of PD-L1 in BGC-823 and MGC-803 and their corresponding LSD1-deleted cell-derived exosomes. **f** Expression of PD-L1 in B-EXO from cells treated with or without GSK2879552 and ORY1001. **g** Expression of PD-L1 in B-EXO & B KO-EXO conjugated with 5 μm beads analyzed by flow cytometry. **h** Expression of PD-L1 in exosomes from MGC-803 cells (M-EXO) and MGC-803 LSD1 KO cells (M KO-EXO) conjugated with 5 μm beads analyzed by flow cytometry. **i** Concentration of B-EXO and B KO-EXO. **j** Expression of PD-L1 in BGC-823 or BGC-823 LSD1 KO cells treated with or without 10 μM GW4869 for 24 h. The data are presented as the mean ± SD; *n* = 3; n.s, no significance, *P < 0.05; two-tailed unpaired Student’s t-test

Previous research has reported that exosomal PD-L1 could also exert immunosuppressive function [28, 48]. PD-L1 is known to be secreted using exosomes as vehicles, which may be regulated by LSD1. Based on this inference, exosomes from GC cells isolated by differential ultracentrifugation as described previously [49] were subjected to SEM and NTA. The results showed that sizes of GC cell-derived exosomes ranged from 30 to 200 nm (Fig. 4a and b). Additional analysis using exosomes purified with sucrose gradient centrifugation suggested that the exosomes markers CD63, ALG-2 interacting protein X (ALIX) and CD9 traveled in the 20%-40% sucrose fractions, and PD-L1 co-localized with these exosome markers (Fig. 4c). The results indicated that PD-L1 was packaged into exosomes from GC cells, and PD-L1 accumulated in exosomes derived from several human GC cell lines (Fig. 4d). To further confirm that the PD-L1 in exosomes was from cancer cells but not from other components, exosome secretion inhibitor GW4869 was used to inhibit the secretion of extracellular vesicles, and there was an obvious reduction of exosomal secretion when exosomes were purified from the same number of cells (Supplementary Fig. 4a) and significant accumulation of membrane PD-L1 (Supplementary Fig. 4b) as well as total PD-L1 (Supplementary Fig. 4c) was observed when BGC-823 cells were exposed to GW4869. Additional data suggested that exosomal PD-L1 was found to be decreased when LSD1 was deleted genetically (Fig. 4e) and pharmacologically (Fig. 4f) in inhibited BGC-823 and MGC-803 cells, indicating the potential role of LSD1 in positively regulating the accumulation of exosomal PD-L1. Moreover, exosomes collected using magnetic beads-based method from LSD1-deleted BGC-823 and MGC-803 cells also harbored less PD-L1 than exosomes from wild-type cells (Fig. 4g and h). Meanwhile, the concentration of GC cell-derived exosomes was decreased when LSD1 was abrogated (Fig. 4i). Therefore, LSD1 KO was deemed to maintain membrane PD-L1 as well as decrease exosomes secretion, and there is a possibility that inhibiting exosomes secretion could rescue this effect. BGC-823 cells were treated with GW4869, and the membrane PD-L1 was quantified. As previously discovered, abrogation of LSD1 can reduce the amount of total PD-L1 (Fig. 3d and f) but maintain the membrane PD-L1 (Supplementary Fig. 3a and 3b). Inhibition of exosome secretion using GW4869 can induce the accumulation of membrane PD-L1 in BGC-823 cells, and GW4869 can also re-induce the accumulation of membrane PD-L1 in LSD1-deleted BGC-823 cells (Fig. 4j). Taken together, these data identified that PD-L1 existed in GC cell-derived exosomes, and LSD1 abrogation decreased the amount of total cellular PD-L1 but maintained membrane PD-L1 as well as decreased exosomes secretion and accumulation of exosomal PD-L1.

### LSD1 deletion reverses the direct T-cell response suppression of GC-derived exosomal PD-L1

**Fig. 5:**
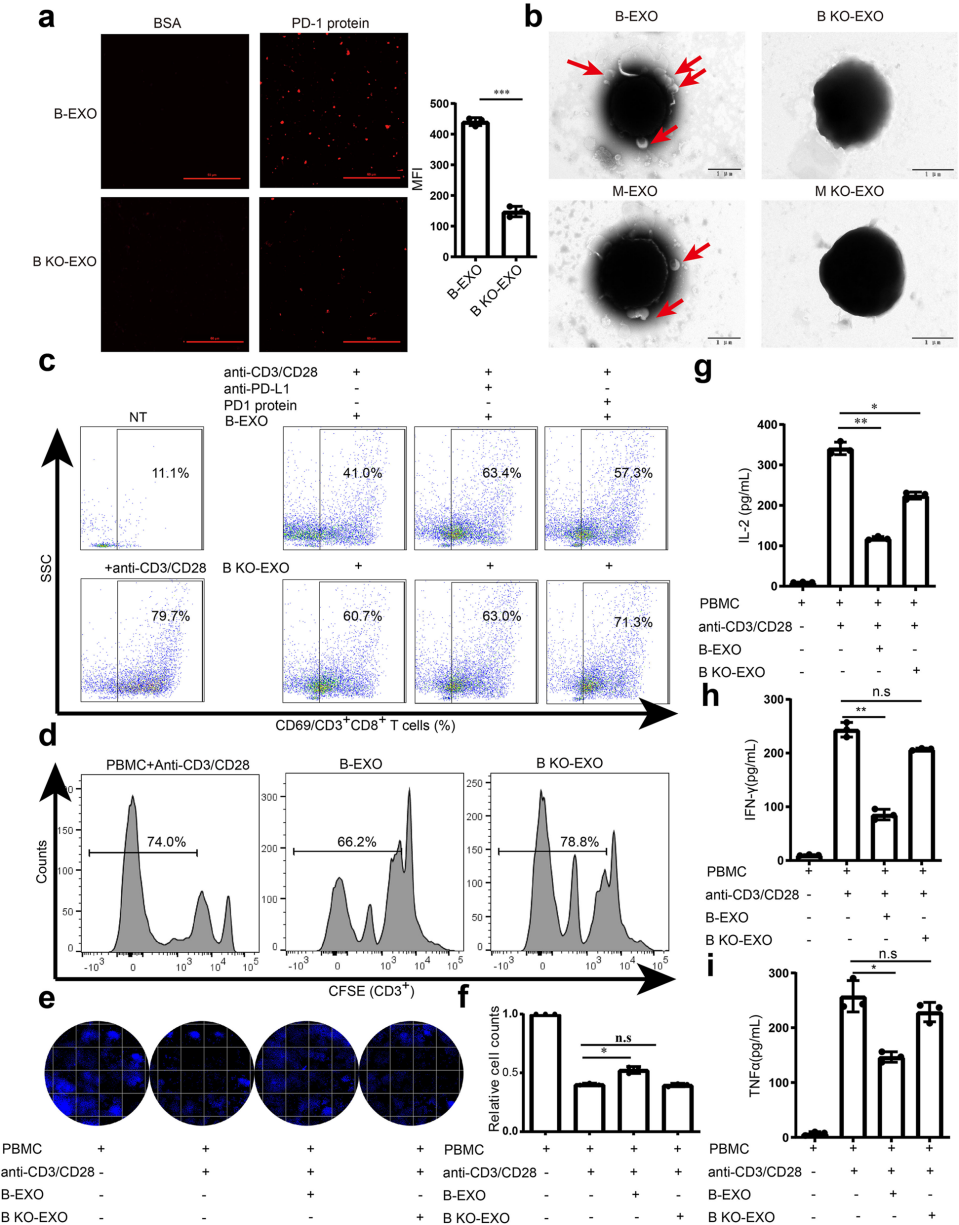
LSD1 deletion reverses the direct T-cell response suppression of GC-derived exosomal PD-L1. **a** Representative confocal images of PKH26 stained B-EXO and B KO-EXO with wells coated with recombinant PD-1. Scale bar = 50 μm. **b** SEM images of the binding of T cells to B-EXO/M-EXO or B KO-EXO/M KO-EXO. Scale bar = 1 μm. **c **Expression of CD69 in CD8 + T cells incubated with B-EXO or B KO-EXO, respectively. NT indicates no treatment with anti-CD3/CD28 beads. **d** T-cell proliferation in anti-CD3/CD28-stimulated PBMC incubated with B-EXO or B KO-EXO. **e** and **f** Cell survival of BGC-823 cells co-incubated with anti-CD3/CD28 beads-activated PBMC in the presence of B-EXO or B KO-EXO. Representative images are shown on the left (**e**), quantification is provided on the right (**f**). **g-i** ELISA analysis of IL-2 (**g**), IFN-γ (**h**), and TNFα (**i**) concentration in anti-CD3/CD28-stimulated PBMC in the presence of B-EXO or B KO-EXO. The data are presented as the mean ± SD; *n* = 3; n.s, no significance, * *P* < 0.05; ** *P* < 0.01; *** *P* < 0.001; two-tailed unpaired Student’s t-test

To ensure that the function of exosomal PD-L1 was retained, PD-1/PD-L1 binding assay was performed to detect the binding ability of exosomal PD-L1 to PD-1. Exosomes from GC cells labeled with PKH26 was found to bind to wells coated with recombinant PD-1, and this binding could be abrogated significantly when LSD1 was deleted in BGC-823 cells (Fig. 5a) [35]. Consistent with this, SEM image of T cells incubated with GC cell-derived exosomes also validated that GC cell-derived exosomes could directly bind to T cells, and LSD1 KO eliminated this interaction (Fig. 5b). In the tumor microenvironment, cancer cells are known to overexpress PD-L1 and its function by binding to PD-1 to inhibit T-cell receptor (TCR)-related pathways and immunologic function. To determine whether LSD1 can regulate the exosomal PD-L1-mediated immunosuppressive effect via the TCR pathway, PBMCs from healthy human donors were activated by anti-CD3/CD28 beads and used for a T-cell stimulation model [11] via co-incubation with B-EXO, and the activation and proliferation of T cells were determined. As represented by CD69, an activation marker of CD8^+^ T cells, anti-CD3/CD28 beads greatly increased CD69 expression [35], and there was obvious downregulation of CD69 in CD8^+^ T cells when treated with B-EXO, while B KO-EXO or blocking the effect of exosomal PD-L1 with a PD-L1 antibody or PD-1 recombinant rescued the suppressed expression of CD69 in CD8^+^ T cells (Fig. 5c). Consistently, T-cell proliferation was decreased in the presence of B-EXO but not when LSD1 was deleted, indicating that the immunosuppressive function of GC cell-derived exosomes was removed by LSD1 KO (Fig. 5d), which is in agreement with the expression level of PD-L1 in exosomes. To further confirm that the exosomes could modulate the GC cell-killing capability of T cells, activated PBMCs were co-cultured with GC cells and treated with exosomes derived from BGC-823 cells in the presence or absence of LSD1. Results suggested that exosomes could reduce T-cell killing to keep the GC cells alive, while B KO-EXO had no effect on T-cell killing due to minimal expression of PD-L1 (Fig. 5e and f). At the same time, secretion of IL-2 (Fig. 5g), IFN-γ (Fig. 5h) and TNFα (Fig. 5i), three main reactive cytokines secreted during T-cell activation, can also be inhibited by B-EXO and rescued by B KO-EXO. These findings consistently demonstrated that exosomes derived from GC cells could directly inhibit TCR-mediated T-cell activation depending on LSD1-regulated exosomal PD-L1.

### LSD1 deletion reverses the inhibitory effect of GC cell-derived exosomes in T-cell response by influencing the expression of PD-L1 in target cells

**Fig. 6:**
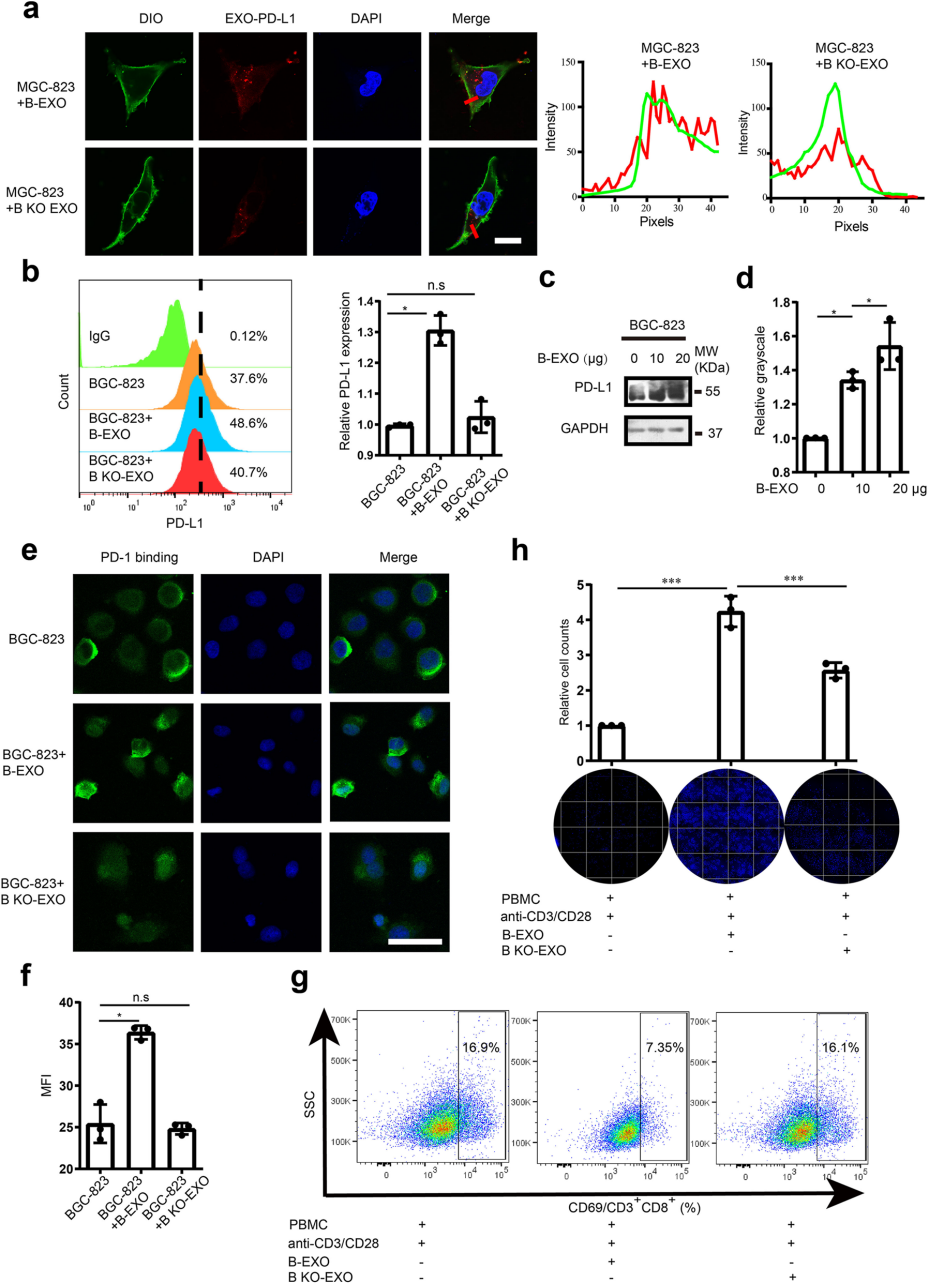
LSD1 deletion reverses the inhibitory effect of GC cell-derived exosomes in T-cell response by influencing the expression of PD-L1 in target cells. **a** Confocal image of 20 μg/ml B-EXO or B KO-EXO that were co-incubated with MGC-803 cells for 24 h. Exosomes were stained with PKH26, followed by membrane dye DIO and nucleus dye DAPI staining. Representative confocal images are shown on the left, whereas quantification is shown on the right. Scale bar = 20 µm. **b-d** Membrane (**b**) and total expression (**c** and **d**) of PD-L1 in BGC-823 cell lines when incubated with B-EXO or B KO-EXO. **e** Confocal images of recombinant PD-1-Fc binding to BGC-823 cell-PD-L1 when cells were incubated with B-EXO or B KO-EXO. Cells were incubated with anti-rabbit Alexa Fluor 488 dye conjugated antibody. Scale bar = 50 μm. **f **Mean fluorescence intensity (MFI) of PD-1-Fc binding when cells were incubated with B-EXO or B KO-EXO. **g** Expression of CD69 in CD8^+^ T-cell when cells were incubated with B-EXO or B KO-EXO. **h** Cell survival of BGC-823 cells in anti-CD3/CD28-stimulated PBMC when incubated with B-EXO or B KO-EXO. The data are presented as the mean ± SD; *n* = 3; n.s, no significance, * *P* < 0.05 *** *P* < 0.001, two-tailed unpaired Student’s t-test

Cancer cells derived exosomes are key players of cell-to-cell communication in the tumor microenvironment [50]. While GC cell-derived exosomes could directly inhibit TCR-mediated T-cell activation, whether GC cell-derived exosomes could deliver the immunosuppressive protein PD-L1 to other cells and the role of LSD1 in this process were still unclear [31]. To explore the transportation function of GC-derived exosomes to other cancer cells, B-EXO and B KO-EXO were stained with fluorescent PD-L1 antibody and incubated with GC cell line MGC-803, and the exosomal PD-L1 was shown to be transported into target GC cells (Fig. 6a). As expected, B-EXO could induce the accumulation of membrane PD-L1 in target cells, while there was no significant PD-L1 accumulation for BGC-823 cells treated with B KO-EXO (Fig. 6b); a similar effect was seen on the total PD-L1 (Fig. 6c and d). Therefore, there was significantly increased binding of PD-1 to the cell surface when treated with B-EXO, while the binding of PD-1 on cells treated with B KO-EXO was kept constant (Fig. 6e and f and Supplementary Fig. 5a & 5b). Accordingly, the expression of CD69 in CD3^+^CD8^+^ T cells was decreased when the activated T cells were co-incubated with BGC-823 cells treated with B-EXO, but not in the group of cells treated with B KO-EXO (Fig. 6g). Phenotypic analysis with the aid of T-cell killing analysis also suggested that GC cells treated with B-EXO intended to escape from T-cell killing, while B KO-EXO had no significant effect on the sensitivity of cancer cells to T-cell killing (Fig. 6h). These results demonstrated that PD-L1 in exosomes could be transported into other cancer cells, thereby inducing immune escape of tumor cells from T cells, while LSD1 deletion decreased the immunosuppressive function of GC cell-derived exosomes, which highlighted LSD1 as a potential immunosuppressive factor for tumor immunotherapy.

### Exosomes from LSD1-abrogated GC cells promotes T cell-mediated tumor immunity through exosomal PD-L1 in vivo

**Fig. 7:**
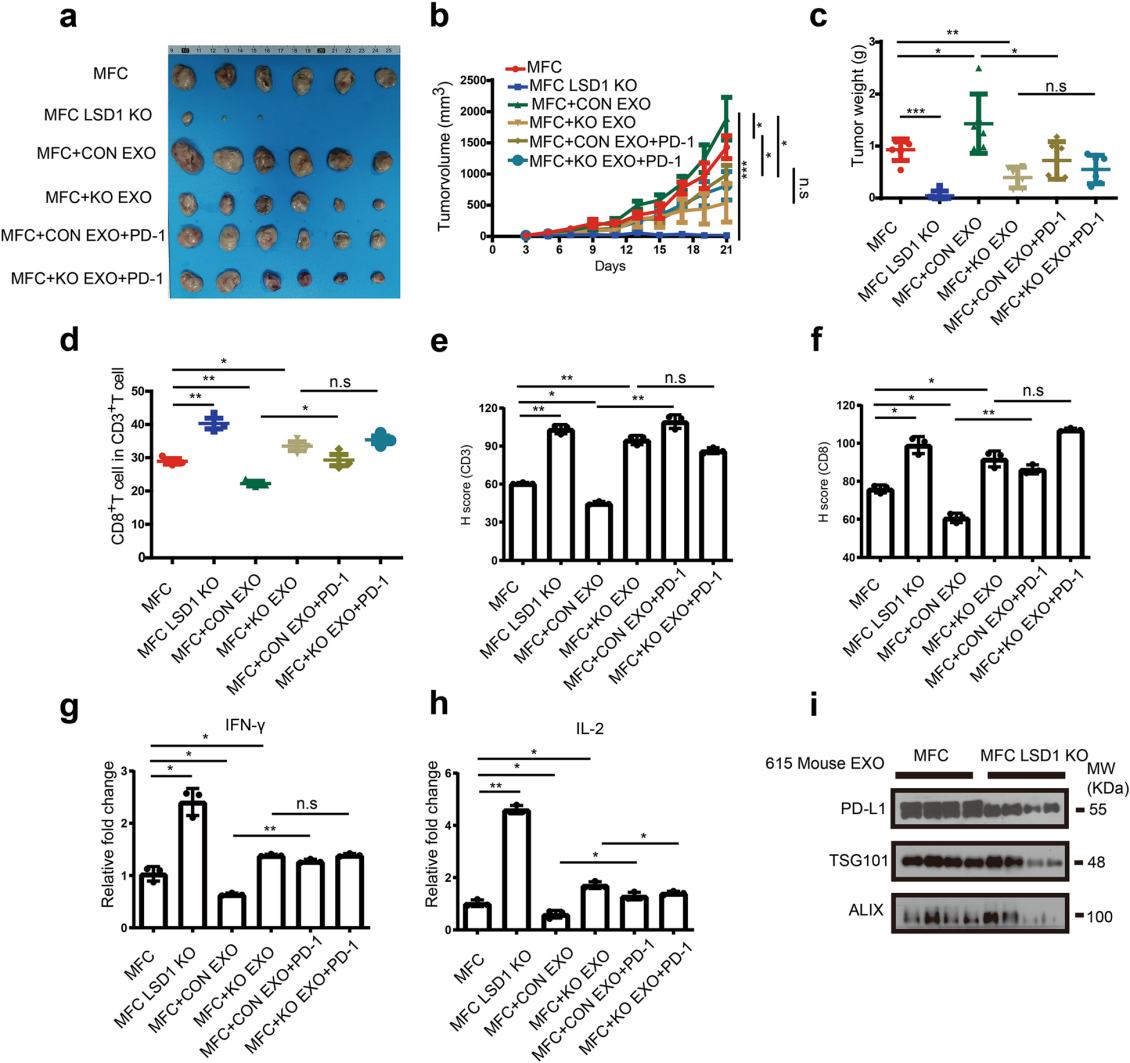
Exosomes from LSD1-abrogated GC cells promoted T-cell mediated tumor immunity through exosomal PD-L1 in vivo. **a-c** Tumor sizes (**a**), growth curve (**b**), tumor weight (**c**) of MFC cells in 615 mice treated with CON EXO or KO EXO in the presence of PD-1-recombinant or not (The data are presented as the mean ± SD, *n* = 6). **d** Tumor-infiltrating CD8 + T cell ratio in CD3 + T cells of MFC cells in 615 mice treated with CON EXO or KO EXO in the presence of PD-1-recombinant or not (The data are presented as the mean ± SD, *n* = 3). **e** and **f** Expression of CD3 and CD8 of MFC cells in 615 mice treated with CON EXO or KO EXO in the presence of PD-1-recombinant or not (The data are presented as the mean ± SD, *n* = 3). **g** and **h** IL-2 (**g**) and IFN-γ (**h**) levels in 615 mice tissues in different groups. Scale bar = 600 μm (The data are presented as the mean ± SD, *n* = 3). **i** Expression of PD-L1 in plasma exosomes of 615 mice treated with MFC cells in the presence or absence of LSD1. The data are presented as the mean ± SD; n.s, no significance, * *P* < 0.05; ** *P* < 0.01; *** *P* < 0.001, two-tailed unpaired Student’s t-test

Given that GC cell-derived exosomes suppressed T-cell activation in vitro, a confirmatory in vivo experiment was performed. Consistent with human GC cells, LSD1 inhibitor GSK2879552 could downregulate PD-L1 expression in MFC cells (Supplementary Fig. 6a) and LSD1 deletion could reduce the expression of exosomal PD-L1 (Supplementary Fig. 6b) as well as LSD1 inhibitors GSK2879552 and ORY1001 (Supplementary Fig. 6c). Therefore, a GC model using 615 mice bearing MFC cells was employed to explore whether GC cell-derived exosomes could regulate tumor progress in vivo. Consistent with our initial findings, LSD1 KO significantly inhibited tumor growth in the 615 mice. To verify whether deletion of LSD1 can promote the tumor immune response by decreasing exosomal PD-L1, exosomes from MFC and MFC LSD1 KO cells (CON EXO and KO EXO, respectively, for short) incubated with or without PD-1 to block PD-L1 were injected into tumors of mice bearing MFC cells for 21 days, and results suggested that tumors of mice injected with exosomes from MFC cells (MFC + CON EXO group) were significantly larger than those with untreated tumors (MFC group), while tumors of mice injected with exosomes from MFC LSD1 KO cells (MFC + KO EXO group) were much smaller than these two groups (Fig. 7a-c). Furthermore, when the exosomes from MFC and MFC LSD1 were incubated with PD-1 in order to block exosomal PD-L1 (MFC + CON EXO + PD-1 group & MFC + KO EXO + PD-1 group), tumor volume and tumor weight were not significantly different among MFC + CON EXO + PD-1 group, MFC + KO EXO + PD-1 group, and MFC + KO EXO group (Fig. 7a-c). These findings led us to hypothesize that treatment with exosomes inhibited CD8^+^ T-cell infiltration in tumors to some extent. Compared to the MFC group, the proportion of CD8^+^ T cells in MFC + CON EXO group was increased significantly, while LSD1 KO cell-derived exosomes promoted the proportion of CD8^+^ T cells (Fig. 7d). On the other hand, when the exosomal PD-L1 was blocked by a PD-1 recombinant, proportion of CD8^+^ T cells in MFC + CON EXO + PD-1 group was higher than that in MFC + CON EXO group (Fig. 7d). Meanwhile, there was no significant difference in the proportion of CD8^+^ T cells between MFC + KO EXO and MFC + KO EXO + PD-1 groups (Fig. 7d). Consistent results were also obtained for the tumor-infiltrating CD3 and CD8 expression in these tumors (Fig. 7e and f, Supplementary Fig. 6d) as well as the amount of cytokines IL-2 and IFN-γ (Fig. 7g and 7h). Moreover, expression of exosomal PD-L1 from plasma of mice bearing MFC LSD1 KO cells was much less than that from plasma of mice bearing MFC cells (Fig. 7i), suggesting that LSD1 has the potential to regulate the global immune response through exosomal PD-L1. Collectively, these results indicated that LSD1 deletion can suppress the proliferation of MFC cells in a syngeneic GC model. Meanwhile, while relying on the existence of LSD1 in the donor cells, exosomes can regulate MFC cell proliferation with distinct roles depending on exosomal PD-L1-mediated T-cell immunity in vivo.

## Discussion

Immunotherapies have exhibited excellent clinical benefits with the progression of checkpoint targeting therapy in cancers. PD-L1, a functional ligand of PD-1, is exploited by tumors to attenuate antitumor immunity and escape from the immune system, thereby facilitating tumor growth. In the previous study, an LSD1 inhibitor was reported to promote tumor immunity through PD-L1 in melanoma and breast cancer [22, 23]. Here, based on the distinct function of LSD1 in T cell-deficient and normal mice (Fig. 1c), we supposed that LSD1 can affect T-cell infiltration in GC, and LSD1-abrogated cells were identified to be more sensitive to T-cell killing. Additional database and clinical tissue-based analyses showed that LSD1 was correlated with PD-L1-mediated T-cell infiltration in GC. In an in-depth study, LSD1 deletion was found to reduce PD-L1 expression and maintain membrane PD-L1 level by reducing the secretion of PD-L1, accompanied by decreased number of multivesicular bodies as well as low expressions of intracellular membrane trafficking TSG101 and RAB11 in GC cells, which drove us to explore the role of LSD1 in exosomal PD-L1. The role of LSD1 regulation of TSG101 and RAB11 remains a question. Certainly, there are other factors that can regulate exosomal PD-L1 secretion that have not been identified and need to be investigated.

Next, LSD1 abrogation was characterized to decrease the amount of exosomal PD-L1, while exosomal PD-L1 directly inhibited T-cell response by increasing the expression of PD-L1 in target GC cells. Hence, LSD1 was confirmed to inhibit T-cell response through exosomal PD-L1, providing strong evidence for the significant contribution of LSD1 in T-cell immunity. In vivo study also provided evidence that exosomes derived from LSD1 in GC cells can promote tumor growth, while exosomes derived from LSD1-deleted GC cells inhibited tumor growth in an exosomal PD-L1-dependent manner. Nevertheless, since LSD1 abrogation in MFC cells almost completely inhibited tumor growth but not exosomes from LSD1-deleted MFC cells, we strongly deem that other regulatory mechanisms still exist for LSD1 in tumor immunity (Fig. 7).

In summary, our findings showed that PD-L1 was identified as a cargo in GC cell-derived exosomes and GC cells can maintain cell membrane PD-L1 by reducing the secretion of exosomal PD-L1 when LSD1 was abrogated. Meanwhile, LSD1 deletion can restore the killing function of T cells in the microenvironment of GC by decreasing the amount of PD-L1 in exosomes as well as by inhibiting PD-L1 transportation to other cancer cells using exosomes as vehicles, thereby offsetting its immunosuppressive function. All of these results indicate a new mechanism by which LSD1 may suppress tumor immunity in GC and provide a new strategy for immunotherapy of GC using LSD1 as a target.

## Conclusion

In summary, LSD1 deletion was found to suppress tumor growth by enhancing T-cell activity through reducing the accumulation of PD-L1 in exosomes, while the membrane PD-L1 was kept constant in GC cells. More than that, using exosomes as vehicles, LSD1 obstructed T-cell response of other cancer cells through exosomal PD-L1 while LSD1 deletion restored T-cell functions. These findings reveal a new pathway by which LSD1 regulates T-cell immunity through exosomal PD-L1 and provide a new strategy for immunotherapy of GC using LSD1 as a target.

## Supplementary Information

**Additional file 1:** **Supplementary Figure 1. **LSD1 KO inhibits tumor growth bymodulating T cells in GC. **a **Correlation analysis of LSD1 with indicated immune cell infiltration in GC by TIMER2.0. **b** Correlation analysis of LSD1 with CD3D, CD3G and CD3E in GC using TCGA database (*n*=544). **c **Expression of LSD1 in BGC-823 and MGC-803 cells with or without LSD1 knocked out using sgRNA. **d** Expression of LSD1 in MFC with or without LSD1 knocked out using sgRNA. **e** and **f** Body weight curves of 615 (**e**) and BALB/c nude (**f**) mice bearing MFC cells in the presence of LSD1 or not (*n*=6). All data are representative of three independently performed experiments.

**Additional file 2:** **Supplementary Figure 2. **Expression of CXCL9, CXCL10 and PD-L1 in GC tissues. **a **Expression of CXCL9, CXCL10 and CD8 in 145 GC tissues. Scales bar = 100 µm. **b **Expression of LSD1 and PD-L1 in 36 paired GC tissues and their corresponding adjacent normal tissues. All data are representative of three independently performed experiments.

**Additional file 3:** **Supplementary Figure 3. **Inhibition of LSD1 does not affect cell membrane PD-L1 expression in GC cells. **a** Expression of PD-L1 in the presence or absence of LSD1 inhibitor GSK2879552 at indicated time. **b** Expression of membrane PD-L1 in the presence or absence of LSD1 inhibitor GSK2879552 for 5 days in BGC-823 cells. **c** Expression of membrane PD-L1 in BGC-823 and MGC-803 with or without LSD1 knocked out. **d** Expression of PD-L1 in BGC-823 cells with or without LSD1 knocked out in the presence of 30 μM CHX at indicated time. **e** Expression of PD-L1 in BGC-823 cells with or without LSD1 knocked out in the presence of 20 μM CQ as indicated. **f** Expression of RAB11 in BGC-823 and MGC-803 cells in the presence of LSD1 or not. **g** Confocal images and quantitative results of PD-L1(red), RAB11 (green) and nucleus (blue) in MGC-803 cells in the presence of LSD1 or not. Scales bar = 20 µm. **h** Confocal images and quantitative results of PD-L1(green), TSG101 (red) and nucleus (blue) in MGC-803 cells in the presence of LSD1 or not. Scales bar, 20 µm. All data are representative of three independently performed experiments

**Additional file 4:** **Supplementary Figure 4. **GW4869 downregulates exosomal PD-L1 in GC cells. **a** Expression of PD-L1, CD63 and ALIX expression in B-EXO from cells in the presence of 10 μM GW4869 or not in the same number of cells. **b** and **c** Expression of membrane PD-L1 (**b**) and total PD-L1 (**c**) in BGC-823 cells treated with GW4869 at indicated concentration. All data are representative of three independently performed experiments.

**Additional file 5:** **Supplementary Figure 5. **Exosomes from LSD1 containing GC cells promote PD-1 binding to recipient cells. **a** Confocal images of recombinant PD-1-Fc binding to PD-L1 in MGC-803 cells when cells were incubated with B-EXO or B KO-EXO. Cells were incubated with anti-rabbit Alexa Fluor 488 dye conjugated antibody. Scale bar, 50 μm. **b** MFI of PD-1-Fc binding when cells were incubated with B-EXO or B KO-EXO. *n* = 3 biological replicates; mean ± S.D; n.s, no significance, * *P* < 0.05, two-tailed unpaired Student’s *t*-test).

**Additional file 6:** **Supplementary Figure 6. **LSD1 abrogation suppresses exosomal PD-L1 in MFC cells and promoted T cell mediated tumor immunity *in vivo* **a** Expression of PD-L1 expression in MFC cells treated with LSD1 inhibitor GSK2879552 for 5 days. **b** Expression of PD-L1 in CON EXO and KO EXO. **c** Expression of PD-L1 in MFC cells derived exosomes in the presence of GSK2879552, ORY1001 or not. All data are representative of three independently performed experiments. **d** Expression of CD3 and CD8 of MFC cells in 615 mice treatment with CON EXO or KO EXO, as well as PD-1 recombinant protein blocking exosomes groups. Scale bar, 600 μm.

## Data Availability

All of the data obtained and/or analyzed during the current study are available from the corresponding authors upon reasonable request.

## Abbreviations

LSD1: Histone lysine-specific demethylase 1
GC: Gastric cancer
PD-L1: Programmed cell death 1 ligand 1
IHC: Immunohistochemistry
TEM: Transmission electronic microscopy
NTA: Nanoparticle tracking analysis
ELISA: Enzyme-linked immunosorbent assay
KO: Knockout
CoREST: Corepressor for RE1-silencing transcription factor
NK: Natural killer
PD-1: Programmed cell death 1
FDA: Food and Drug Administration
STR: Short tandem repeat
FBS: Fetal bovine serum
PBS: Phosphate buffer solution
BCA: Bicinchoninic acid
SDS-PAGE: Sodium dodecyl sulfate–polyacrylamide gel electrophoresis
DIO: 3,3’-Dioctadecyloxacarbocyanine perchlorate
DAPI: 4',6-Diamidino-2-phenylindole
CFSE: 5, 6-Carboxyfluorescein diacetate, succinimidyl ester
IL-2: Interleukin 2
IFN-γ: Interferon-gamma
TNFα: Tumor necrosis factor α
qRT-PCR: Real-time quantitative polymerase chain reaction
RAB11: Ras-related GTP-binding protein 11
TSG101: Tumor susceptibility gene 101 protein
EDTA: Ethylene diamine tetraacetic acid
TCGA: The Cancer Genome Atlas
CXCL9: C-X-C motif chemokine 9
CXCL10: C-X-C motif chemokine 10
CHX: Cycloheximide
CQ: Chloroquine
CD63: CD63 molecule
ALIX: ALG-2 interacting protein X
CD9: CD9 molecule
TCR: T cell receptor
PBMCs: Peripheral blood mononuclear cells
MFI: Mean fluorescence intensity
