# Supplementary figures and images for: LSD1 deletion decreases exosomal PD-L1 and restores T-cell response in gastric cancer
Source: Mol Cancer. 2022 Mar 16;21:75. doi: 10.1186/s12943-022-01557-1 (PMC8925194; doi:10.1186/s12943-022-01557-1)

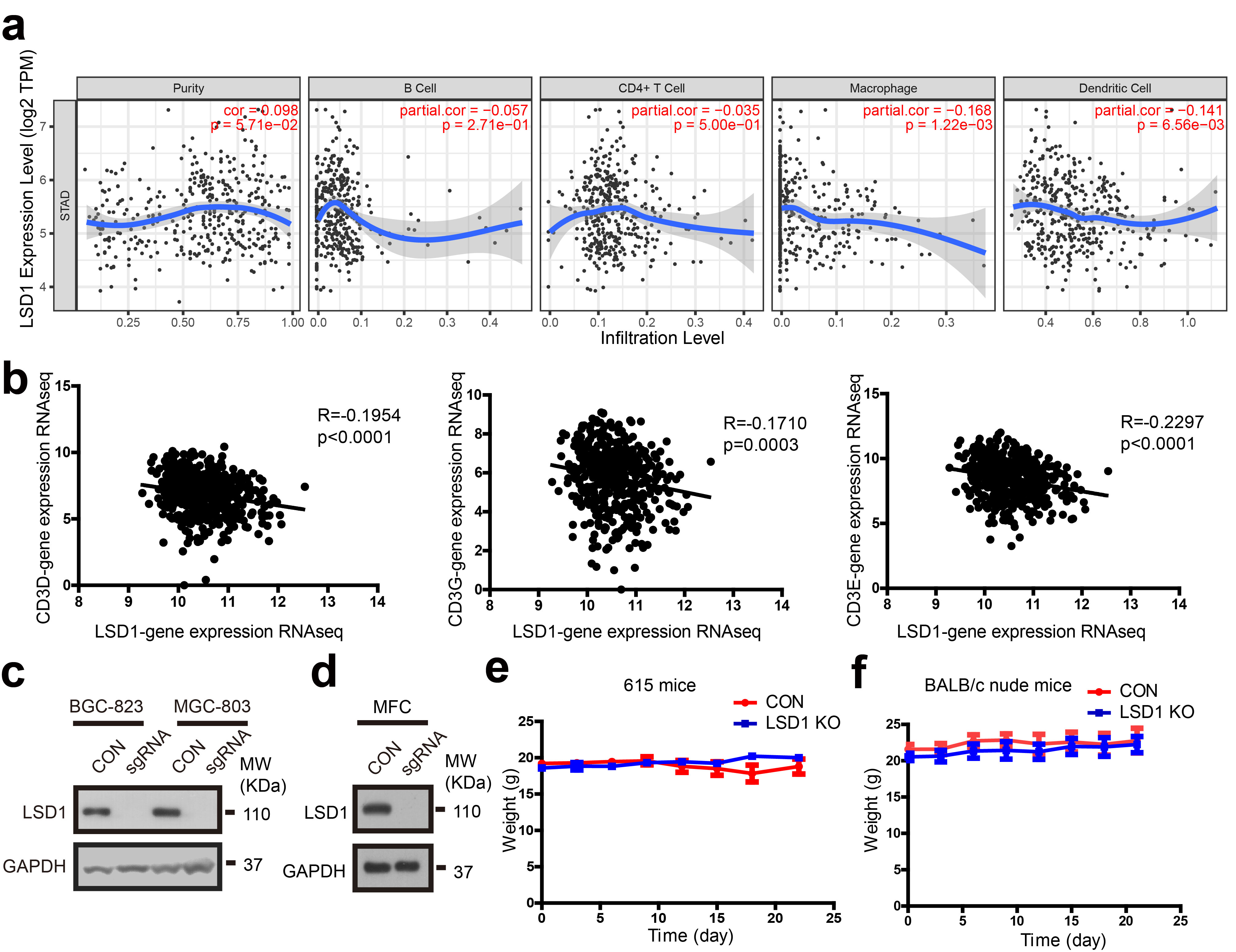

Supplement: Supplementary file 1 — Additional file 1: Supplementary Figure 1. LSD1 KO inhibits tumor growth bymodulating T cells in GC. a Correlation analysis of LSD1 with indicated immune cell infiltration in GC by TIMER2.0. b Correlation analysis of LSD1 with CD3D, CD3G and CD3E in GC using TCGA database (n=544). c Expression of LSD1 in BGC-823 and MGC-803 cells with or without LSD1 knocked out using sgRNA. d Expression of LSD1 in MFC with or without LSD1 knocked out using sgRNA. e and f Body weight curves of 615 (e) and BALB/c nude (f) mice bearing MFC cells in the presence of LSD1 or not (n=6). All data are representative of three independently performed experiments. [file 12943_2022_1557_MOESM1_ESM.jpg]

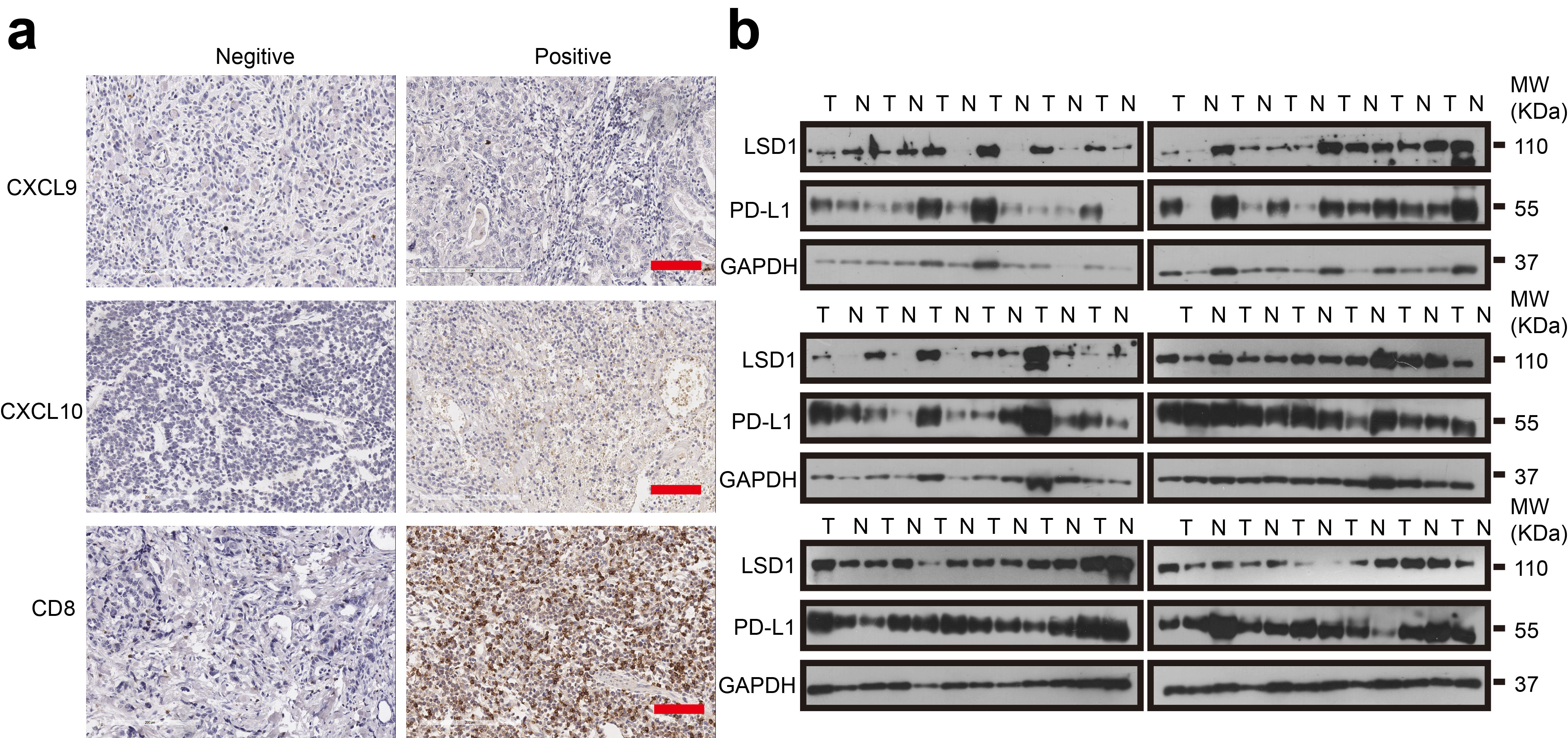

Supplement: Supplementary file 2 — Additional file 2: Supplementary Figure 2. Expression of CXCL9, CXCL10 and PD-L1 in GC tissues. a Expression of CXCL9, CXCL10 and CD8 in 145 GC tissues. Scales bar = 100 µm. b Expression of LSD1 and PD-L1 in 36 paired GC tissues and their corresponding adjacent normal tissues. All data are representative of three independently performed experiments. [file 12943_2022_1557_MOESM2_ESM.jpg]

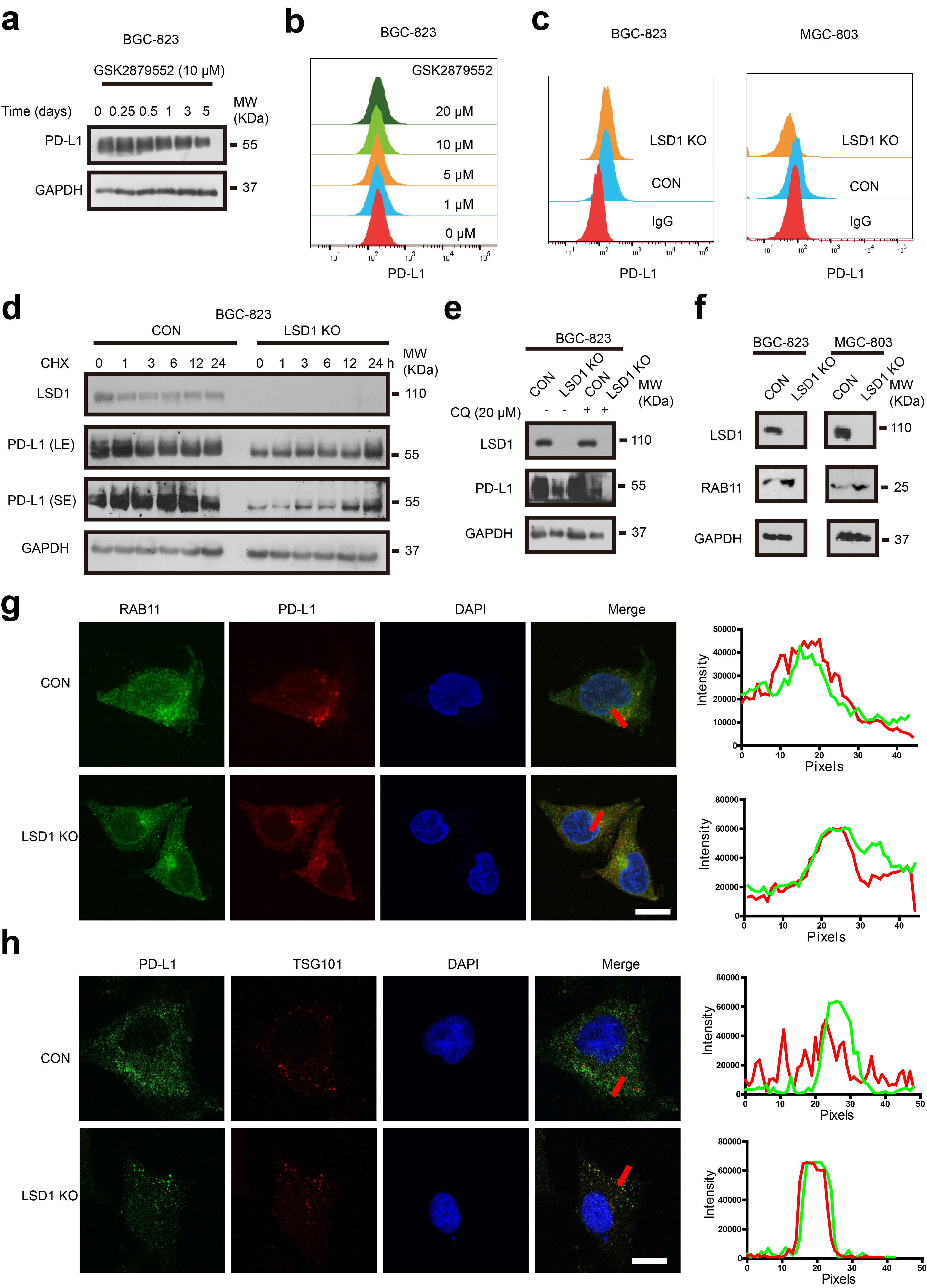

Supplement: Supplementary file 3 — Additional file 3: Supplementary Figure 3. Inhibition of LSD1 does not affect cell membrane PD-L1 expression in GC cells. a Expression of PD-L1 in the presence or absence of LSD1 inhibitor GSK2879552 at indicated time. b Expression of membrane PD-L1 in the presence or absence of LSD1 inhibitor GSK2879552 for 5 days in BGC-823 cells. c Expression of membrane PD-L1 in BGC-823 and MGC-803 with or without LSD1 knocked out. d Expression of PD-L1 in BGC-823 cells with or without LSD1 knocked out in the presence of 30 μM CHX at indicated time. e Expression of PD-L1 in BGC-823 cells with or without LSD1 knocked out in the presence of 20 μM CQ as indicated. f Expression of RAB11 in BGC-823 and MGC-803 cells in the presence of LSD1 or not. g Confocal images and quantitative results of PD-L1(red), RAB11 (green) and nucleus (blue) in MGC-803 cells in the presence of LSD1 or not. Scales bar = 20 µm. h Confocal images and quantitative results of PD-L1(green), TSG101 (red) and nucleus (blue) in MGC-803 cells in the presence of LSD1 or not. Scales bar, 20 µm. All data are representative of three independently performed experiments [file 12943_2022_1557_MOESM3_ESM.jpg]

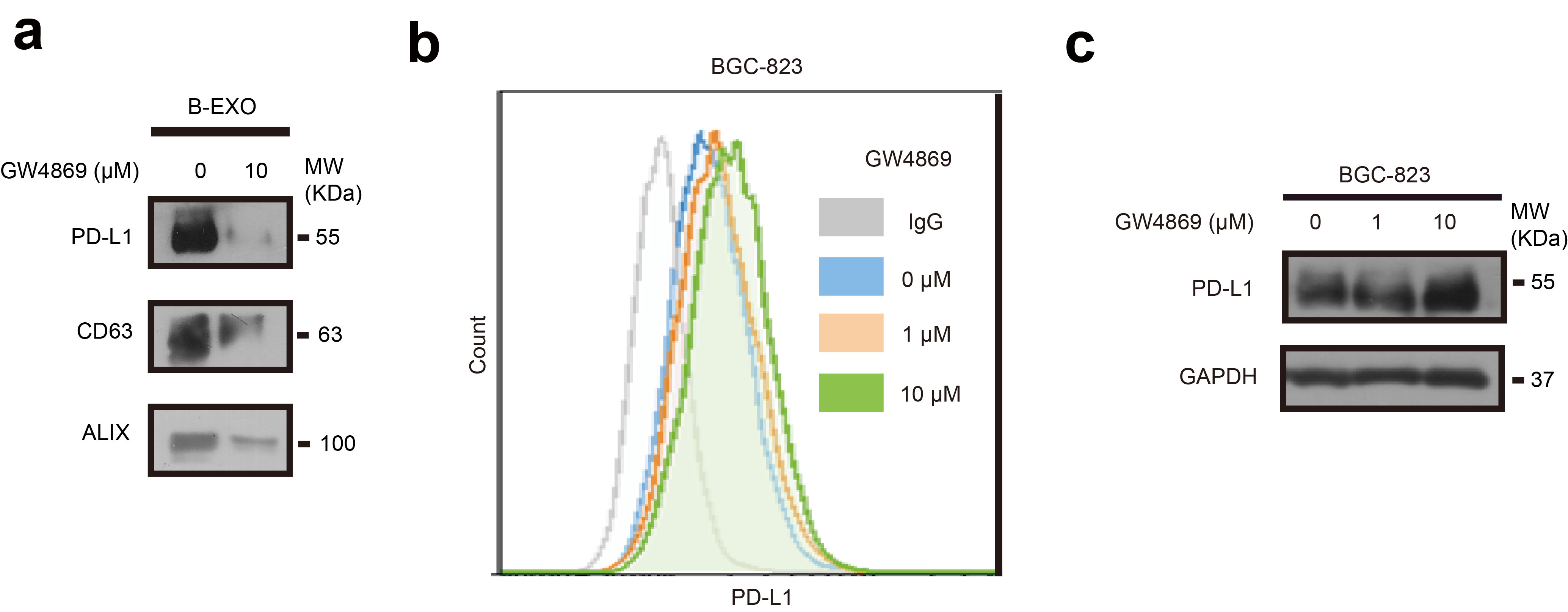

Supplement: Supplementary file 4 — Additional file 4: Supplementary Figure 4. GW4869 downregulates exosomal PD-L1 in GC cells. a Expression of PD-L1, CD63 and ALIX expression in B-EXO from cells in the presence of 10 μM GW4869 or not in the same number of cells. b and c Expression of membrane PD-L1 (b) and total PD-L1 (c) in BGC-823 cells treated with GW4869 at indicated concentration. All data are representative of three independently performed experiments. [file 12943_2022_1557_MOESM4_ESM.jpg]

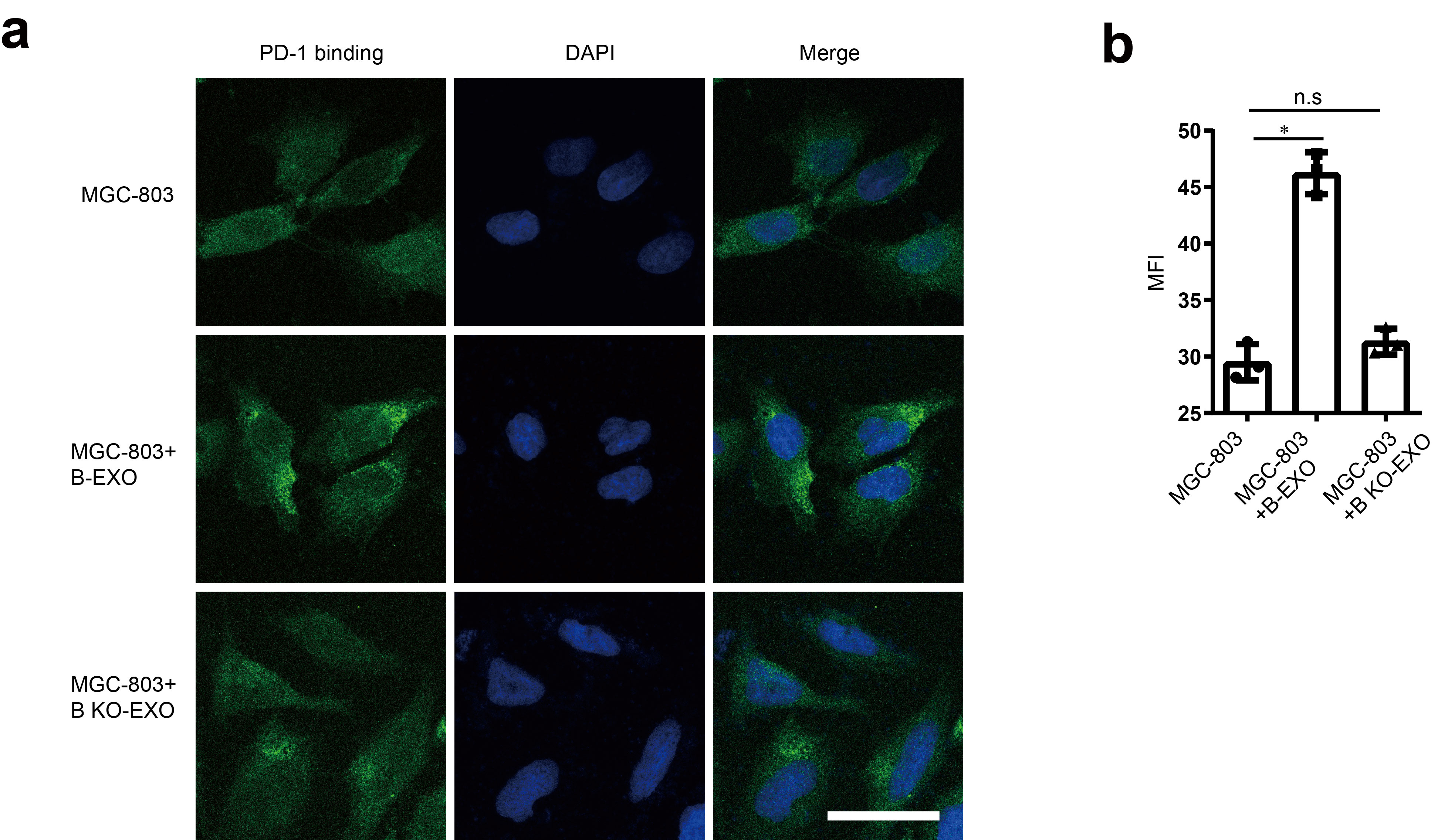

Supplement: Supplementary file 5 — Additional file 5: Supplementary Figure 5. Exosomes from LSD1 containing GC cells promote PD-1 binding to recipient cells. a Confocal images of recombinant PD-1-Fc binding to PD-L1 in MGC-803 cells when cells were incubated with B-EXO or B KO-EXO. Cells were incubated with anti-rabbit Alexa Fluor 488 dye conjugated antibody. Scale bar, 50 μm. b MFI of PD-1-Fc binding when cells were incubated with B-EXO or B KO-EXO. n = 3 biological replicates; mean ± S.D; n.s, no significance, * P < 0.05, two-tailed unpaired Student’s t-test). [file 12943_2022_1557_MOESM5_ESM.jpg]

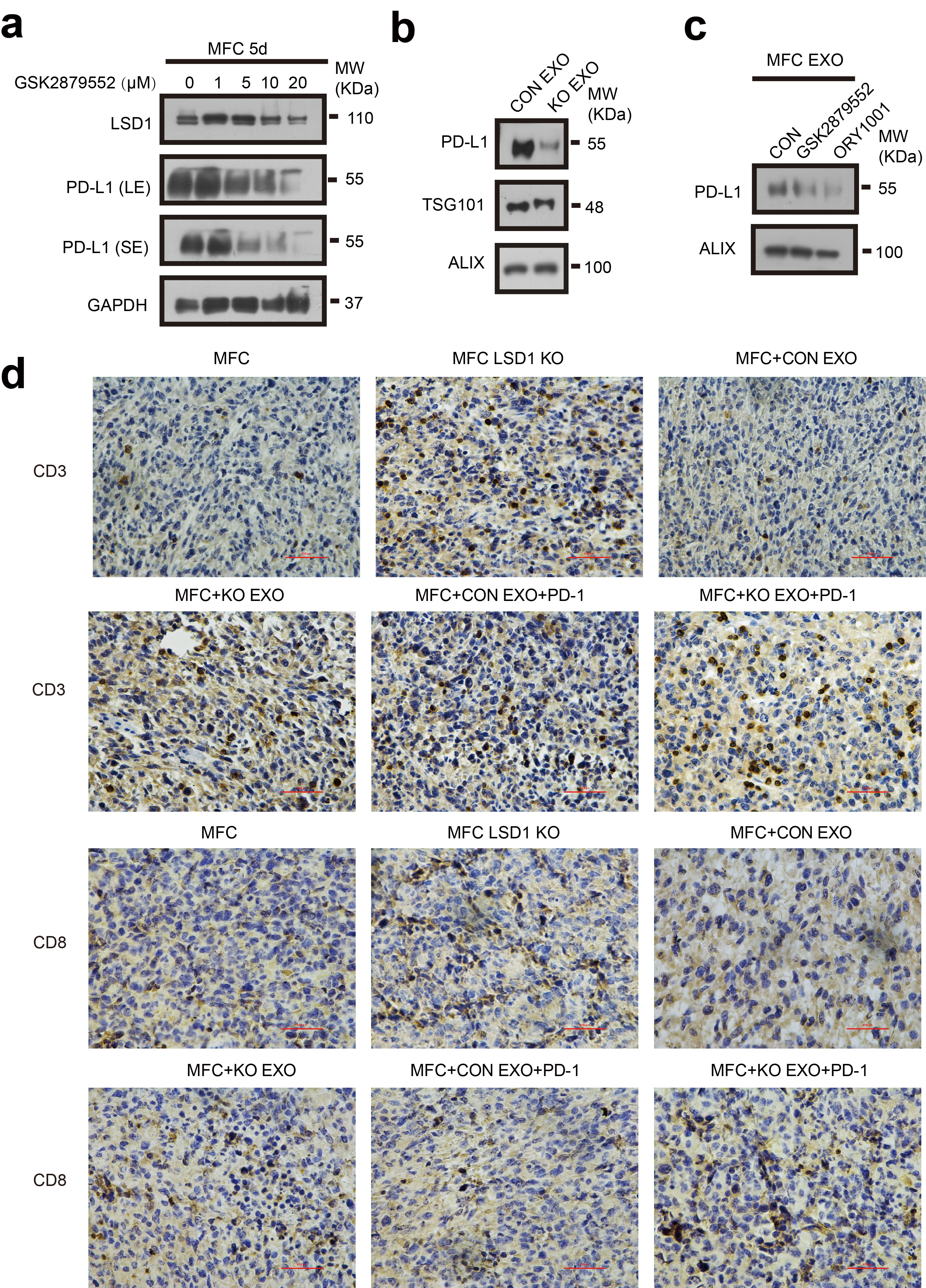

Supplement: Supplementary file 6 — Additional file 6: Supplementary Figure 6. LSD1 abrogation suppresses exosomal PD-L1 in MFC cells and promoted T cell mediated tumor immunity in vivo a Expression of PD-L1 expression in MFC cells treated with LSD1 inhibitor GSK2879552 for 5 days. b Expression of PD-L1 in CON EXO and KO EXO. c Expression of PD-L1 in MFC cells derived exosomes in the presence of GSK2879552, ORY1001 or not. All data are representative of three independently performed experiments. d Expression of CD3 and CD8 of MFC cells in 615 mice treatment with CON EXO or KO EXO, as well as PD-1 recombinant protein blocking exosomes groups. Scale bar, 600 μm. [file 12943_2022_1557_MOESM6_ESM.jpg]
